# Development and Validation of Multivariable Machine‐Learning Models for the Prediction of Multisystemic Inflammatory Syndrome Outcomes in Latin American Children

**DOI:** 10.1111/apa.70290

**Published:** 2025-09-01

**Authors:** Danilo Buonsenso, Luca Mastrantoni, Rolando Ulloa‐Gutierrez, Jimena García‐Silva, Gabriela Ivankovich‐Escoto, Marco A. Yamazaki‐Nakashimada, Enrique Faugier‐Fuentes, Olguita del Águila, German Camacho‐Moreno, Dora Estripeaut, Iván F. Gutiérrez‐Tobar, Adriana H. Tremoulet, The REKAMLATINA‐3 MIS‐C Study Group Investigators, Kathia Luciani, Mariana Fabi, Graciela Espada, Marcela Álvarez, Martha I. Álvarez‐Olmos, Jaime Fernández‐Sarmiento, Paola Pérez‐Camacho, Saulo Duarte‐Passos, Maria C Cervi, Edwin M Cantillano, Beatriz A Llamas‐Guillén, Mónica Velásquez‐Méndez, Patricia Saltigeral‐Simental, Enrique Chacon‐Cruz, Miguel García‐Domínguez, Karla L Borjas Aguilar, Ana V Villarreal‐Treviño, Sandra Beltrán, Andrea Gatica, Fernanda Cofré, Virgen Gómez, Heloisa HS Marques, Nadina E Rubio‐Pérez, Luis M Garrido‐García, Luisa B Gámez‐González, Carlos Daza, Genara M Santana‐Chalas, Humberto García‐Aguilar, Elmer H Zapata‐Yarlequé, Lucila Martínez‐Medina, Adán Cuatecontzi‐Romero, Shirley Cuan, Alejandro Díaz, Adrián Collia, Lorena Franco, Elizabeth Assandri, Adriana Díaz‐Maldonado, Elizabeth Castaño, Ximena Norero, Raúl Esquivel, Jacqueline Levy, Katherina Miranda, Scarlett Sinesterra, Manuel Alvarado, Aldo Campos, Adriana Yock‐Corrales, Alejandra Soriano‐Fallas, Kattia Camacho‐Badilla, Jéssica Gómez‐Vargas, Lourdes Dueñas, Pilar Guarnizo, Manuel Huertas‐Quiñones, Diana C Medina‐Ramos, Sara I. Aguilera‐Martínez, Verónica Morales‐Burton, Jaime Patiño, Lina M Sandoval‐Calle, Rolando Andrés Paternina‐de la Ossa, Diana López‐Gallegos, Juan Pablo Rojas, Elizabeth Moreno, Mónica Pujadas, Maria C Pirez, Fernando García‐Rodríguez, Martha Márquez‐Aguirre, Tirza de León, Jesús G Montaño‐Durón, Manuel Munaico‐Abanto, Daniel Jarovsky, Maynor G Bravo‐López, Alejandro Ellis, Antonio González‐Mata, Mario Melgar, Antonio Luévanos‐Velázquez

**Affiliations:** ^1^ Department of Woman and Child Health and Public Health Fondazione Policlinico Universitario A. Gemelli IRCCS Rome Italy; ^2^ Area Pediatrica, Dipartimento di Scienze della Vita e Sanità Pubblica Università Cattolica del Sacro Cuore Rome Italy; ^3^ Medical Oncology Università Cattolica del Sacro Cuore Rome Italy; ^4^ Servicio de Aislamiento Pediátrico, Hospital Nacional de Niños “Dr. Carlos Sáenz Herrera” Caja Costarricense de Seguro Social (CCSS) San José Costa Rica; ^5^ Universidad de Ciencias Médicas (UCIMED) e Instituto de Investigación en Ciencias Médicas UCIMED (IICIMED) San José Costa Rica; ^6^ Academia Nacional de Medicina de Costa Rica (ACANAMED) San José Costa Rica; ^7^ Departamento de Pediatría, Hospital Universitario “José Eleuterio González” Universidad Autónoma de Nuevo León Monterrey Nuevo León Mexico; ^8^ Servicio de Inmunología y Reumatología Pediátrica, Hospital Nacional de Niños “Dr. Carlos Sáenz Herrera” Caja Costarricense de Seguro Social (CCSS) San José Costa Rica; ^9^ Departamento de Inmunología Clínica Instituto Nacional de Pediatría Ciudad de México Mexico; ^10^ Servicio de Reumatología Hospital Infantil de México Federico Gómez Ciudad de México Mexico; ^11^ Unidad de Infectología Pediátrica Hospital Nacional Edgardo Rebagliati Martins Lima Peru; ^12^ Unidad de Infectología Pediátrica, HOMI, Fundación Hospital Pediátrico La Misericordia & Departamento de Pediatría, Facultad de Medicina Universidad Nacional de Colombia Bogotá Colombia; ^13^ Servicio de Infectología Hospital del Niño Dr. José Renán Esquivel Ciudad de Panamá Panama; ^14^ Servicio de Infectología Clínica Infantil Colsubsidio Bogotá Colombia; ^15^ Department of Pediatrics University of California San Diego (UCSD) & Rady Children's Hospital San Diego California USA

**Keywords:** COVID‐19, Latin America, MIS‐C, prediction models, web‐app

## Abstract

**Aim:**

We aimed to develop and test machine learning algorithms for the prediction of severe outcomes associated with MIS‐C.

**Method:**

An observational ambispective cohort study was conducted including children aged from 1 month to 18 years old in 84 hospitals from the REKAMLATINA (Red de la Enfermedad de Kawasaki en America Latina) network diagnosed with MIS‐C from 1st January 2020 to 31st June 2022. Multiple models were developed to predict four main outcomes: paediatric intensive care unit (PICU) admission, need for inotropes, need for mechanical ventilation, and death. Performance measures were accuracy for PICU admission, inotropes use and mechanical ventilation, and the area under the receiver operating characteristic curve (AUROC) for death. Variable contribution was analysed using Shapley Additive Explanations (SHAP) values.

**Results:**

We included 1303 children with a diagnosis of MIS‐C. The model for the prediction of PICU admission (random forest [RF]) reached an accuracy of 0.80 (95% CI: 0.76–0.84), the model for inotrope use (RF) an accuracy of 0.86 (95% CI: 0.82–0.90), the model for mechanical ventilation (histogram‐based gradient boosting [HBGB]) an accuracy of 0.84 (95% CI 0.80–0.88), and the model for death (RF) reached an AUROC of 0.85 (95% CI 0.77–0.93).

**Conclusions:**

We developed and validated machine learning models for the prediction of MIS‐C related outcomes that can help clinicians risk stratify patients to identify those most likely to have a severe outcome from MIS‐C.

**Summary**

- MIS‐C can lead to severe outcomes in children, and early identification of high‐risk patients is crucial for timely interventions.
- In 1303 children from 84 Latin American hospitals, machine learning models accurately predicted PICU admission (0.80), inotrope use (0.86), mechanical ventilation (0.84), and death (AUROC 0.85).
- These validated models can support clinicians in risk stratification, potentially improving patient outcomes and guiding resource allocation in MIS‐C care.

## 1 Introduction

Multisystem inflammatory syndrome in children (MIS‐C) has been the most severe acute complication of the SARS‐CoV‐2 infection, described soon after the beginning of the COVID‐19 pandemic [1, 2]. MIS‐C is now considered a post‐acute complication of previous SARS‐CoV‐2 exposure or infection, and is characterised by a hyperinflammatory state and a clinical presentation that resembles Kawasaki disease [2] although the conditions may differ immunologically and by clinical and laboratory characteristics [3]. Nevertheless, as many signs and symptoms overlap, the two conditions may be confused. A common feature of MIS‐C, which is less frequently found in Kawasaki disease, is the presence of shock and gastrointestinal symptoms [4, 5], sometimes so severe that it mimics an acute surgical abdomen [6]. Given the presence of shock, most children are managed in the paediatric intensive care unit (PICU) and may require inotropic support. However, not all children may present to the first medical evaluation with a severe picture requiring PICU support but can unfavourably evolve over time. Deaths have also been reported [7].

Although several studies have been published on MIS‐C, and some have attempted to define predictors of poor outcomes [8, 9, 10], most of these studies have focused on only one specific outcome. However, PICU admission, or even the use of mechanical ventilation, can be biased by local practices, while other outcomes like impaired systolic function or death can be more objective. Nevertheless, since these outcomes are relatively rare, most studies have not had enough power. The lack of clear predictors of several different clinically relevant outcomes has practical implications, particularly from a therapeutic perspective. Currently, the best treatment for MIS‐C is unclear, with most centers using intravenous immunoglobulins with or without steroids [11, 12]. However, several studies have also documented benefits from the use of biological agents. Theoretically, a model that can define several negative outcomes may guide clinicians to start more aggressive treatments earlier in high‐risk children. In addition, such a model may serve as the basis to develop similar ones for other inflammatory conditions, including Kawasaki disease.

This study addressed these gaps with three primary aims. First, we sought to develop and validate different machine learning models capable of predicting multiple clinically relevant outcomes of MIS‐C in a large cohort of Latin American children, using easily accessible parameters readily available during initial clinical evaluation. Second, adhering to the principles of explainable machine learning, we identified the most critical clinical predictors associated with each outcome to assist physicians in risk stratification. Third, we developed an interactive web app to promote the routine use of our models by healthcare professionals as a clinical decision support tool.

## 2 Methods

The Latin American Kawasaki Disease Network (REKAMLATINA—Red de la Enfermedad de Kawasaki en America Latina) is a multinational, multicenter research network that was initially established for Kawasaki disease. This network, composed of many of the main paediatric and general referral hospitals in Latin America, maintains a detailed observational registry of Kawasaki disease cases. Since the COVID‐19 pandemic, the registry has been expanded to include MIS‐C cases from 84 participating centres across 16 Latin American countries: Argentina, Bolivia, Brazil, Chile, Colombia, Costa Rica, Cuba, Dominican Republic, Ecuador, El Salvador, Guatemala, Honduras, Mexico, Panama, Paraguay, Peru, Puerto Rico, Uruguay, and Venezuela [13, 14].

The aim of this new study was to develop a model based on common clinical and laboratory findings that predicts several outcomes of MIS‐C in children, including PICU admission, use of inotropics, mechanical ventilation, and death. This study was reported according to the TRIPOD‐AI statement (Table S2).

### 2.1 Inclusion Criteria

Patients who met the Centers for Disease Control definition of MIS‐C and were diagnosed from 1 August 2020 to 31 June 2022 were included in the study. According to clinical criteria, the illness should have been characterised by all of the following: fever (≥ 38°C), clinical severity requiring hospitalisation, C‐reactive protein increase (CRP, ≥ 3.0 mg/dL), new onset in at least two categories (cardiac involvement, mucocutaneous involvement, shock, gastrointestinal involvement or haematological involvement) and laboratory confirmed SARS‐CoV‐2 infection (positive SARS‐CoV‐2 real‐time reverse‐transcriptase polymerase chain reaction or antibody test during hospitalisation) or an epidemiologic linkage (close contact with a confirmed or probable case of COVID‐19 within the 4 weeks prior to the onset of symptoms) [15].

### 2.2 Measurement of Variables and Endpoints

The Network database includes data on demographics (age, sex, parent‐reported race/ethnicity), clinical signs and symptoms, vital parameters upon admission to the hospital, laboratory test results as well as radiological exams, electrocardiography (ECG) and echocardiographic findings, destination of the patient (PICU or ward), need for inotropic support or mechanical ventilation and duration of support, treatments and response to therapy, complications, and outcome. Further details on how data were collected in the dataset are provided in previous Network studies [16].

For the purpose of this study, we focused on clinical and diagnostic parameters easily available. The following variables were initially considered for the analysis: age (in years), sex, ethnicity (parent‐reported), comorbidities, signs and symptoms (dehydration, cough, conjunctival injection, mucositis, lymphadenopathy, extremity edema, rash, convulsions, shock, abdominal pain, vomiting and diarrhoea), blood tests (complete blood cell count, C‐reactive protein (CRP), erythrocyte sedimentation rate (ESR), procalcitonin, creatinine, liver enzymes, ferritin, pro‐BNP), diagnostics (ECG, echocardiogram including left ventricular function and coronary abnormalities), and therapeutic interventions (intravenous fluids, inotropes, transfusions, intravenous immunoglobulin, aspirin, steroids, antibiotics, antivirals, and immunomodulators). Signs and symptoms were considered as reported by the treating physician. Normal ECG was defined as the lack of arrhythmia, conduction abnormalities, ST changes, QT changes, QRS changes, and normal echocardiography as normal systolic and diastolic function, absence of pleural effusion, and absence of coronary artery abnormalities.

The outcomes were PICU admission, need for inotropic support, mechanical ventilation, and death. Each outcome was considered at any possible time after admission. Outcomes assessors were not blinded given the objective nature of the outcomes.

### 2.3 Statistical Analysis

Clinicopathological characteristics at baseline were described using standard descriptive statistics. Continuous variables were reported as median and interquartile range (IQR) and compared with the Mann–Whitney U test. Categorical variables were reported as frequency and percentage and assessed using a chi‐square test or Fisher's exact test, as appropriate. While no formal sample size calculation was performed in advance, the large number of included patients and the models' performances suggest that an adequate number of observations per variable was reached. Statistical analyses were performed using RStudio (version 4.2.2; RStudio, PBC, Boston, Massachusetts, United States) and Python (version 3.10; Python Software Foundation, Wilmington, Delaware, United States). All hypotheses were two‐sided and *p* < 0.05 was considered statistically significant, except when otherwise specified.

### 2.4 Pre‐Processing

For model development and clinical interpretation, 26 variables were chosen based on domain knowledge, previous studies [10, 11, 17], potential clinical utility and availability. Samples with missing data for at least one of the outcomes and patients with less than 20 predictors available (75%) were excluded. Then, the full cohort was split in a train/validation set using a 70/30 split. Age, fever days, haemoglobin (Hb), platelet count, lymphocytes, C‐reactive protein (CRP), procalcitonin (PCT), creatinine, alanine aminotransferase (ALT), aspartate aminotransferase (AST), ferritin and pro‐BNP were considered as numerical variables while dehydration, cough, conjunctivitis, mucositis, lymphadenopathy, extremities edema, rash, convulsions, shock, belly pain, vomiting, diarrhoea, ECG (normal/altered), and echocardiography (normal/altered) as binary variables. The four endpoints (PICU admission, inotropes use, mechanical ventilation and death) were considered as binary variables, configuring a supervised binary classification task. All categorical variables were encoded using one‐hot encoding. To account for the possible presence of outliers, continuous variables were windsorized at the 5th and 95th percentiles (extreme values below and above the 5th percentile and 95th percentile were replaced with the corresponding percentile) and then standardised using *z*‐scores. Missing data were imputed using median imputation for continuous variables and the most frequent category for binary variables. Sensitivity analyses were conducted using k‐nearest neighbour (KNN) imputation and multivariate imputation by chained equations (MICE).

### 2.5 Model Development

Using the sklearn package, Histogram‐Based Gradient Boosting (HBGB) and Random Forest (RF) algorithm were trained, including in the pipeline hyperparameter tuning with Optuna using 5‐fold cross‐validation and Platt (sigmoid) calibration [18]. Accuracy was used as the target function for PICU admission, inotropic use, and mechanical ventilation, and AUROC for death, due to the imbalance of the outcome frequency. 95% confidence intervals for metrics used were calculated using 2000 bootstrap replicates. Further models were trained using the AutoML framework from the H_2_O package, using a different train/test split to evaluate the consistency of different splitting on the full dataset. This version trains and cross‐validates the following algorithms: XGB, Generalised Linear Models (GLMs: Ridge regression, LASSO regression and Elastic Net), RF, Extremely Randomised Trees, Gradient Boosting Machines (GBM) and densely connected deep neural network (DNN). At the end of the process, additional stacked ensembles are trained using all the base models and only one of the best models from each algorithm family. For each run, the maximum number of models was set to 20, with hyperparameter tuning performed using grid search and five‐fold cross‐validation. For death, upsampling was used to reduce class imbalance. For each outcome, a GLM was also trained to favour variables interpretability.

### 2.6 Model Evaluation and Variable Importance

For each model, we evaluated the confusion matrix, the learning curve, and the performance metrics (AUROC, AUC‐PR, accuracy, precision and recall). The best model for each outcome was chosen based on the cross‐validated performance on the train set. The best models were then evaluated on the validation set. The calibration curve was inspected to evaluate the agreement between observed outcomes and model‐estimated values.

With the aim of favouring model explainability and to enhance the use of the clinical implication of these analyses, we inspected variable importance and partial dependence plots (PDP). Shapley Additive exPlanations (SHAP) permutation explainer beeswarm plot and heatmap for each variable for each of the best models was also evaluated.

### 2.7 Ethics

The REKAMLATINA REDCap database has received institutional review board approval at the University of California, San Diego (La Jolla, CA, USA), and the study was approved at each individual institution enrolling subjects in the REKAMLATINA MIS‐C REDCap database, as well as ethical approval from CENDEISSS (Centro de Desarrollo Estratégico e Información en Saud y Seguridad Social) as required for the leading hospital of the study, Hospital Nacional de Niños “Dr. Carlos Sáenz Herrera”, Caja Costarricense de Seguro Social (CCSS) in San José, Costa Rica.

## 3 Results

### 3.1 Patients' Characteristics

After pre‐processing, we included 1303 of the 1323 children diagnosed with MIS‐C (female 45.5%) with a median age of 6.6 (IQR 2.8–10.6) years. The main demographic characteristics, symptoms, and laboratory findings at admission are reported in Table 1 and Figure S1. The most frequent symptoms were abdominal pain (835, 64.1%), conjunctival injection (793, 60.9%) and rash (773, 59.3%). Shock was reported in 488 (37.5%) patients. Overall, 623 (47.8%) children were admitted to the PICU, 522 (40.1%) required inotropic support, 272 (20.9%) required mechanical ventilation, and 69 (5.3%) died. After the train/test split, 912 children were included in the train set and 391 in the test set. The univariate analyses for demographic, clinical, and laboratory characteristics in patients from the train set grouped according to the main outcomes are detailed in Table S1.

**TABLE 1:** Distribution of symptoms, clinical characteristics and laboratory values at for study population.

	Overall (*n* = 1303)	Train (*n* = 912)	Test (*n* = 391)	Missing
Age, years	6.6 [2.8–10.6]	6.9 [3.0–10.6]	6.0 [2.2–10.6]	0
Sex				0
Female	593 (45.5)	417 (45.7)	176 (45.0)	
Male	710 (54.5)	495 (54.3)	215 (55.0)	
Ethnicity				113
Afro‐American	29 (2.2)	8 (2.0)	21 (2.3)	
Indigenous	10 (0.8)	4 (1.0)	6 (0.7)	
Mestizo	855 (65.6)	247 (63.2)	608 (66.7)	
White	296 (22.7)	85 (21.7)	211 (23.1)	
Clinical characteristics				
Fever days	5.0 [4.0–7.0]	5.0 [4.0–7.0]	5.0 [4.0–7.0]	21
Dehydration	467 (35.8)	338 (37.1)	129 (33.0)	12
Cough	411 (31.5)	288 (31.6)	123 (31.5)	5
Conjunctival injection	793 (60.9)	570 (62.5)	223 (57.0)	4
Oral Erythema	548 (42.1)	400 (43.9)	148 (37.9)	6
Cervical lymphadenopathy	354 (27.2)	248 (27.2)	106 (27.1)	15
Extremity edema	520 (39.9)	370 (40.6)	150 (38.4)	8
Rash	773 (59.3)	546 (59.9)	227 (58.1)	3
Convulsions	54 (4.1)	36 (3.9)	18 (4.6)	9
Shock	488 (37.5)	356 (39.0)	132 (33.8)	4
Abdominal pain	835 (64.1)	588 (64.5)	247 (63.2)	20
Vomiting	728 (55.9)	516 (56.6)	212 (54.2)	1
Diarrhoea	588 (45.1)	413 (45.3)	175 (44.8)	6
Laboratory values				
Haemoglobin, g/dL	11.2 [9.9–12.4]	11.2 [9.9–12.3]	11.1 [9.9–12.5]	0
Platelet count, cells × 10^9^/L	190.0 [110.0–294.8]	186.0 [109.0–287.0]	201.0 [114.0–310.2]	5
Lymphocytes, cells/μL	1240.0 [639.0–2400.0]	1200.0 [636.0–2280.0]	1410.0 [647.5–2788.5]	28
Neutrophils, cells/μL	7755.0 [4097.5–11909.8]	8255.0 [4413.0–11675.0]	7600.0 [3986.8–12100.0]	207
C‐reactive protein, mg/dL	14.0 [6.5–24.8]	14.5 [6.7–25.0]	13.0 [5.9–23.2]	52
ESR, mm/h	38.0 [22.0–59.0]	40.0 [25.0–58.2]	37.0 [21.0–59.0]	
Procalcitonin, ng/mL	3.0 [0.7–13.2]	3.1 [0.8–13.0]	2.5 [0.5–13.5]	647
Creatinine, mg/dL	0.5 [0.3–0.7]	0.5 [0.3–0.7]	0.5 [0.3–0.7]	41
ALT, U/L	39.6 [22.0–78.2]	40.0 [23.1–77.6]	38.0 [21.0–79.8]	35
AST, U/L	43.0 [28.0–80.0]	44.0 [28.0–80.0]	41.5 [27.6–79.0]	33
Ferritin, ng/mL	444.0 [226.0–957.8]	439.0 [218.8–926.4]	450.0 [232.3–996.4]	231
pro‐BNP, pg/mL	1112.0 [279.0–5042.0]	1259.0 [281.5–5264.5]	784.0 [278.0–4376.0]	703
Other exams				
Echocardiography normal	620 (47.6)	425 (46.6)	195 (49.9)	97
ECG normal	607 (46.6)	425 (46.6)	182 (46.5)	496
Outcomes				
PICU admission	623 (47.8)	435 (47.7)	188 (48.1)	0
Inotropes use	522 (40.1)	377 (41.3)	145 (37.1)	0
Mechanic ventilation	272 (20.9)	190 (20.8)	82 (21.0)	0
Death	69 (5.3)	49 (5.4)	20 (5.1)	0

*Note:* Continuous variables are represented as median [IQR]. Categorical variables as *n* (%).

Abbreviations: ALT, alanine aminotransferase; AST, aspartate aminotransferase; ESR, Erythrocyte sedimentation rate; IQR, Interquartile range; PICU, paediatric intensive care unit; proBNP, pro B‐type natriuretic peptide.

### 3.2 Predictive Models for PICU Admission

**FIGURE 1:**
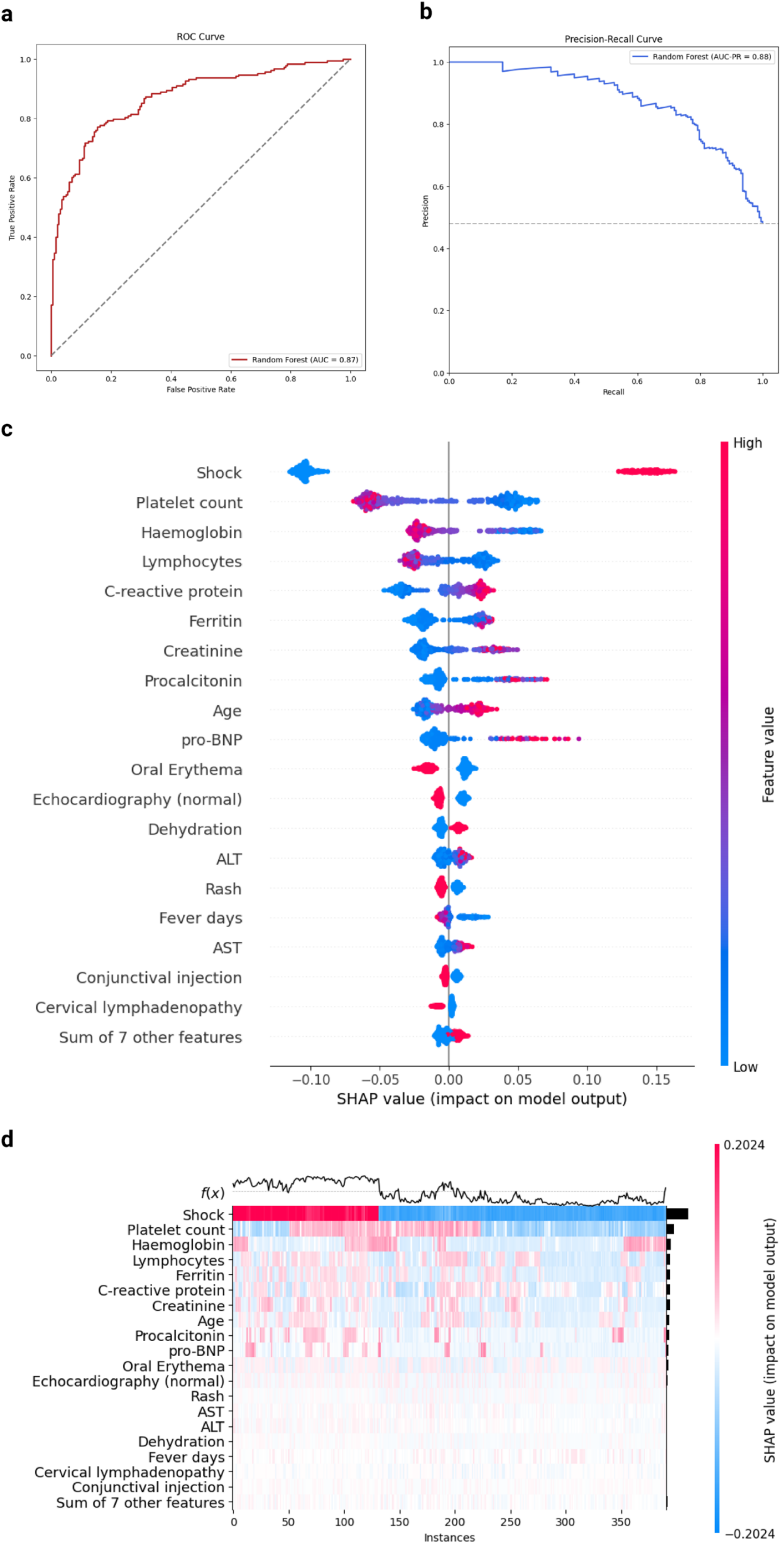
Model evaluation and variable importance for PICU admission. (a) ROC curve. The area under the ROC curve (AUROC) is reported. (b) Precision‐recall curve. The area under the precision‐recall curve (AUC‐PR) is reported. (c) SHAP value for included features. The feature values are presented on the right, with higher values plotted in red and lower values in blue. Positive SHAP value are associated with an increased risk for the outcome, for the corresponding value of the feature. (d) SHAP heatmap for the most important features. The risk function *f*(*x*) in presented on the upper part of the heatmap, with higher values corresponding to higher risk. Higher SHAP values are reported in red while lower SHAP value in blue. Colour intensity is proportional to the absolute SHAP value. PICU, paediatric intensive care unit.

For the prediction of PICU admission, the RF model achieved a mean cross‐validated accuracy on the 5 folds of 0.76 in the training set and a 0.80 (95% CI 0.76–0.84) accuracy on the test set (Figure S2A). The test AUROC was 0.87 (95% CI 0.83–0.90) and the PR‐AUC 0.88 (95% CI 0.84–0.92) (Figure 1A,B). The model showed good calibration (Figure S2B).

In the SHAP analysis, the five variables with the higher absolute contribution were shock, platelets, haemoglobin, lymphocytes, and ferritin. In detail, PICU admission was associated with low Hb levels, low platelet count, and low lymphocytes, as well as elevated CRP, creatinine, procalcitonin, and pro‐BNP. Notably, oral erythema and rash were associated with negative SHAP values (Figure 1C). In the heatmap analysis, whereas shock remained the main determinant, some high‐risk clusters emerged, such as the combination of low Hb levels and low platelet levels, with or without pro‐BNP increase; and the combination of low platelet count with elevated creatinine, CRP, or procalcitonin levels (Figure 1D).

These findings were confirmed in the GLM with an accuracy of 0.78 and an AUROC of 0.84 on the test set, which also highlighted older age and cough as other risk factors. As expected, the presence of a normal echocardiography reduced the risk of PICU admission (Figure S2). In the exploration of PDPs for different models, we observed how the risk tended to increase linearly for a platelet count below 300 × 10^9^/L, lymphocyte values below 2000 mm^3^, and Hb values below 11 mg/dL. A similar trend was also observed for creatinine values above 0.6 mg/dL and ferritin values above 500 ng/mL (Figure S3).

### 3.3 Predictive Models for Inotropes Use

**FIGURE 2:**
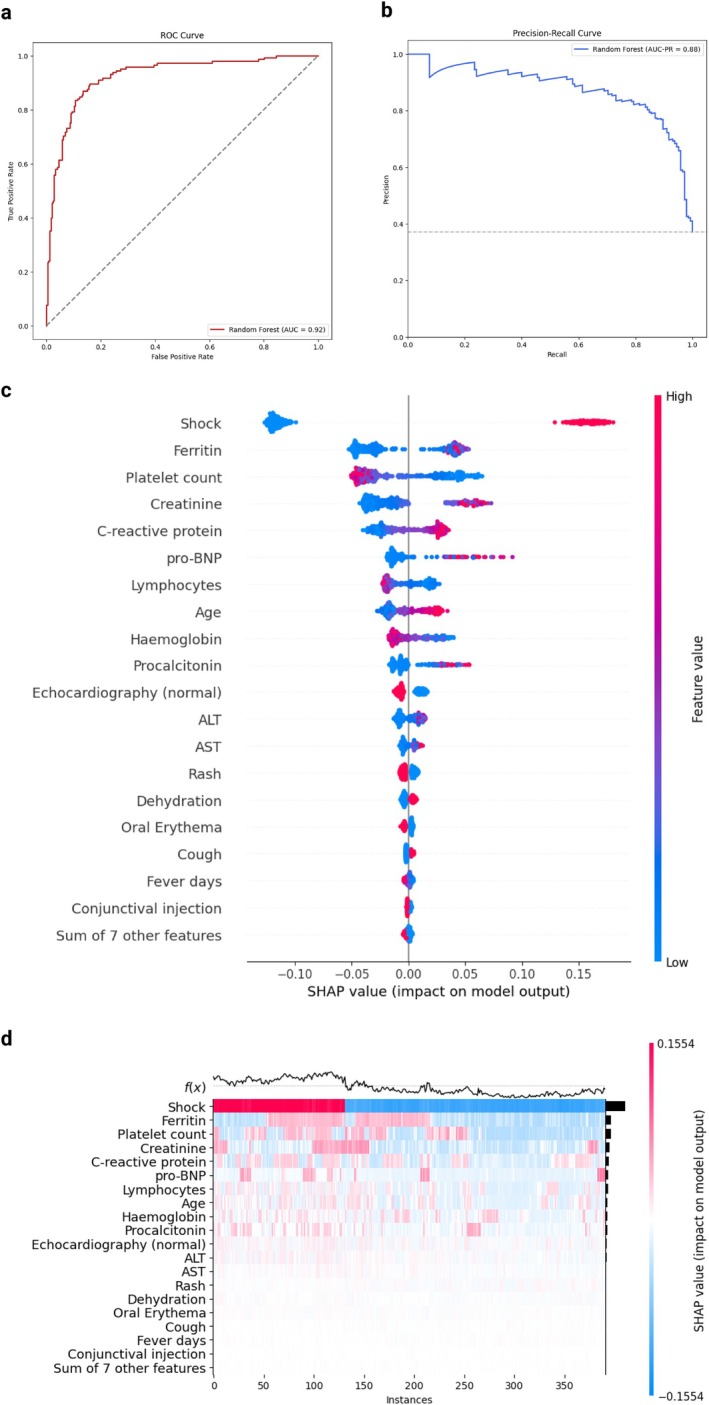
Model evaluation and variable importance for inotropes use. (a) ROC curve. The area under the ROC curve (AUROC) is reported. (b) Precision‐recall curve. The area under the precision‐recall curve (AUC‐PR) is reported. (c) SHAP value for included features. The feature values are presented on the right, with higher values plotted in red and lower values in blue. Positive SHAP value are associated with an increased risk for the outcome, for the corresponding value of the feature. (d) SHAP heatmap for the most important features. The risk function *f*(*x*) in presented on the upper part of the heatmap, with higher values corresponding to higher risk. Higher SHAP values are reported in red while lower SHAP value in blue. Colour intensity is proportional to the absolute SHAP value.

RF model achieved a mean cross‐validated accuracy on the five folds of 0.81 in the training set and a 0.86 (95% CI 0.82–0.90) accuracy on the validation set for the prediction of inotropes use (Figure S4). The test AUROC was 0.92 (95% CI 0.89–0.95) and the PR‐AUC 0.88 (95% CI 0.82.0.93) (Figure 2A,B). The model showed good calibration (Figure S4B).

In the SHAP analysis, the five variables with the higher absolute contribution were shock, ferritin, platelet count, creatinine, and CRP. In detail, elevated ferritin, creatinine, CRP, pro‐BNP, procalcitonin, and ALT/AST, as well as older age, were also associated with positive SHAP values, as well as low platelet count and low lymphocytes. A normal echocardiography was associated with negative SHAP values (Figure 2C). In the heatmap analysis, the combination of an elevated platelet count, ferritin, and creatinine was associated with higher risk, followed by the combination of thrombocytopenia with either increased creatinine or ferritin (Figure 2D).

These findings were confirmed in the GLM (with an accuracy of 0.83 and an AUROC of 0.88 on the test set), where the five variables with the highest importance were shock, CRP, platelet count, creatinine, and seizures (Figure S4). In the exploration of PDPs, we observed how the risk increased similarly to PICU admission for platelet count, creatinine, and Hb. Notably, a much steeper increase in the risk was observed for pro‐BNP and ferritin values above 400 ng/mL (Figure S5).

### 3.4 Predictive Models for Mechanic Ventilation

**FIGURE 3:**
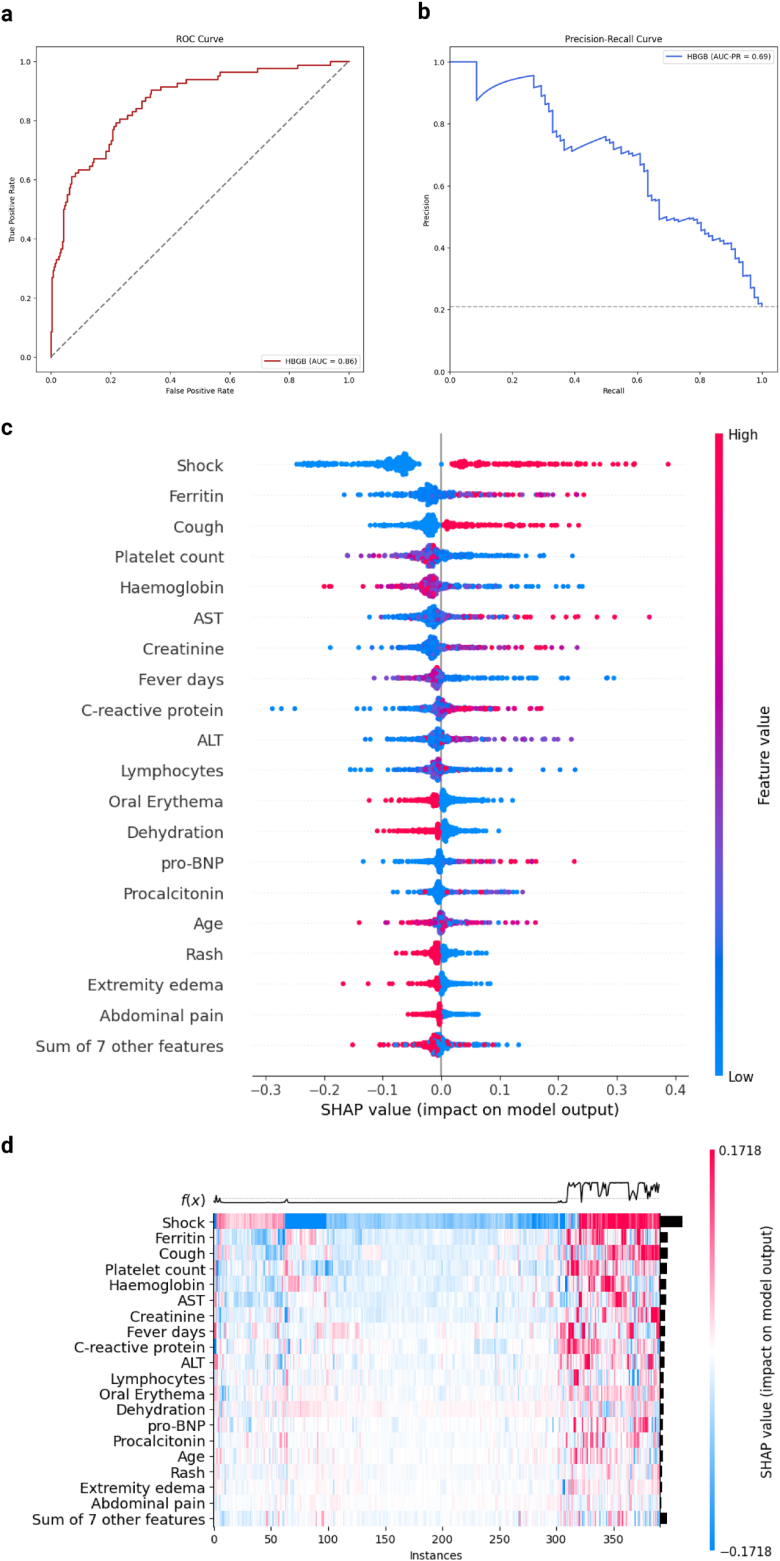
Model evaluation and variable importance for mechanic ventilation. (a) ROC curve. The area under the ROC curve (AUROC) is reported. (b) Precision‐recall curve. The area under the precision‐recall curve (AUC‐PR) is reported. (c) SHAP value for included features. The feature values are presented on the right, with higher values plotted in red and lower values in blue. Positive SHAP value are associated with an increased risk for the outcome, for the corresponding value of the feature. (d) SHAP heatmap for the most important features. The risk function *f*(*x*) in presented on the upper part of the heatmap, with higher values corresponding to higher risk. Higher SHAP values are reported in red while lower SHAP value in blue. Colour intensity is proportional to the absolute SHAP value.

The HBGB model for mechanical ventilation prediction achieved a mean cross‐validated accuracy on the 5 folds of 0.83 in the training set and a 0.84 (95% CI 0.80–0.88) accuracy on the test set (Figure S6). The test AUROC was 0.86 (95% CI 0.82–0.90) and the PR‐AUC was 0.69 (95% CI 0.59–0.78) (Figure 3A). The model showed good calibration (Figure S6).

In the SHAP analysis, the variables with the highest absolute SHAP values were shock, ferritin, cough, platelet count, and Hb. Elevated ferritin, ALT/AST, CRP, and creatinine were also associated with an increased mechanical ventilation risk, as well as low platelet count and Hb (Figure 3C). In the heatmap analysis, the higher‐risk cluster was the combination of cough, low platelet count, and elevated ferritin (Figure 3D).

Variable importance was consistent in the GLM (with an accuracy of 0.84 and an AUROC of 0.85 on the test set) (Figure S6). In the exploration of PDPs, we observed a similar risk increase to PICU admission and inotropes use for Hb, lymphocytes, platelet count, and creatinine, and a step increase in risk for ferritin values above 400 ng/mL (Figure S7).

### 3.5 Predictive Models for Death

**FIGURE 4:**
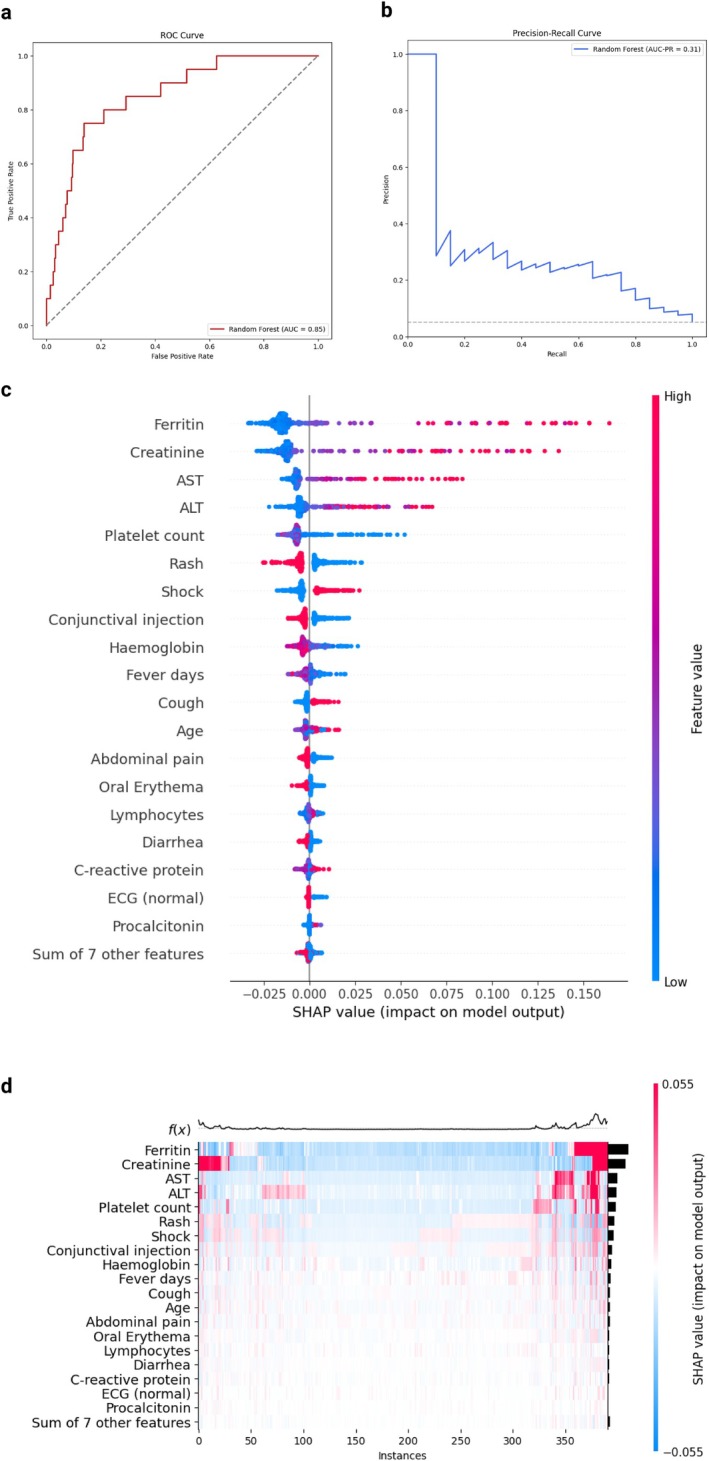
Model evaluation and variable importance for death. (a) ROC curve. The area under the ROC curve (AUROC) is reported. (b) Precision‐recall curve. The area under the precision‐recall curve (AUC‐PR) is reported. (c) SHAP value for included features. The feature values are presented on the right, with higher values plotted in red and lower values in blue. Positive SHAP value are associated with an increased risk for the outcome, for the corresponding value of the feature. (d) SHAP heatmap for the most important features. The risk function *f*(*x*) in presented on the upper part of the heatmap, with higher values corresponding to higher risk. Higher SHAP values are reported in red while lower SHAP value in blue. Colour intensity is proportional to the absolute SHAP value.

For death prediction, the RF model achieved a mean cross‐validated AUROC on the 5 folds of 0.84 in the training set and 0.85 (95% CI 0.77–0.93) on the test set, with a PR‐AUC of 0.31 (Figure 4). The test accuracy was 0.95 (95% CI 0.93–0.97) (Figure S8) and the model showed fair calibration (Figure S8).

In the SHAP analysis, elevated ferritin, elevated creatinine, and elevated ALT/AST were associated with higher SHAP values for death, as well as low platelet count and shock. Rashed and conjunctival injections were associated with negative SHAP values (Figure 4C). Consistently, in the heatmap analysis, the highest risk was associated with the concomitant presence of creatinine, ferritin, and/or transaminase increase (Figure 4D).

In this scenario, the GLM had an accuracy of 0.96 and an AUROC of 0.68 on the test set and a PR‐AUC of 0.16. Creatinine was confirmed to be the most important predictor followed by cough, shock and AST increase (Figure S8C,D). Notably, in the PDP, creatinine values greater than 1.1 mg/dL were associated with an exponential increase in the risk of death. Interestingly, while the absolute contribution of AST was greater than ALT, we observed a step risk increase for AST values above 200 U/L and for ALT values above 50 U/L. A higher risk of death was also observed for platelets below 100 × 10^9^/L and ferritin values above 1000 ng/mL (Figure S9A–F).

### 3.6 Sensitivity Analyses

Sensitivity analyses according to imputation methods yielded consistent results (Table S3).

### 3.7 Model Availability

To improve the clinical availability of these models, the best model for each outcome was included in a virtual application for the early prediction of the outcomes of children based on the aforementioned parameters and it is freely available at https://rekamlatina‐938txe27bue34ehmgdhgtm.streamlit.app/.

## 4 Discussion

To our knowledge, this was the first and largest dataset of children with MIS‐C to be evaluated with multiple advanced models. This led to the development of a virtual application for the early prediction of meaningful outcomes of MIS‐C based on parameters that can be obtained at the time of admission. Such an approach can serve for the early personalised risk stratification of children with MIS‐C, therefore allowing potential personalization of care with intensification of therapy. In addition, our approach may serve as a base for the development of a similar model in other hyperinflammatory conditions, including but not limited to Kawasaki disease.

Although new MIS‐C cases have significantly decreased compared with the early phases of the pandemic [19], this study serves as an example of what can be done with baseline, readily available clinical data to predict disease severity in an inflammatory disorder. Specifically, for the treatment of MIS‐C, several retrospective multinational cohort studies have not been able to determine a definitive benefit of one strategy over another (e.g., intravenous immunoglobulins vs. steroids, or intravenous immunoglobulins +steroids vs. one of the two) [10, 11, 12, 20, 21, 22, 23]. Even in the largest cohorts published, biological agents were rarely used, and critical outcomes were relatively rare, therefore limiting statistical power to compare biologics vs. other treatments in improving the outcomes of MIS‐C [10, 11, 12, 20, 21, 22, 23]. Given all these uncertainties, early recognition of the most‐at‐risk patient may support practitioners in defining when more aggressive therapeutic strategies can be implemented.

Previous studies have mostly attempted to predict ICU admission or death based on simpler multivariate analyses, without providing a tool that might be used clinically [11, 12, 23]. Whereas linear models are widely used and easily interpretable, they often fail in considering nonlinearity and interactions, unless pre‐specified. Machine learning approaches, on the other hand, offer a more flexible and data‐driven alternative. Implementing algorithmic learning procedures, these methods can automatically identify and model nonlinear associations, high‐dimensional interactions, and latent structures without the need to impose strong a priori assumptions regarding the functional form of relationships [24]. Moreover, in line with the principles of explainable artificial intelligence (XAI), machine learning predictions can be supported by variable importance measures that quantify the relative contribution of each predictor to the model's output. Such approaches extend interpretability beyond traditional regression coefficients, enabling clinicians to better assess the relation between individual variables and predicted outcomes. With our approach, we have been able to upload all the models in a virtual channel, freely accessible, producing a real‐time calculator that with simple information, collected at first clinical assessment, is able to predict MIS‐C outcomes. As such, this tool has the potential to be tested in other populations and, if its validity is confirmed in other settings, may have the potential to be used around the globe.

Although the models were based on simple information, they were found to be fairly accurate, particularly for the need for inotropes and mechanical ventilation, which may be the two more objective outcomes, along with death. By contrast, PICU admission can not only be related to the need for objective invasive procedures or monitoring, but also may be influenced by local practice and decisions of the attending physician, as described for other conditions [25]. Conversely, shock and mechanical ventilation are more objective parameters that were accurately predicted by our model soon after the first clinical assessment. For death, given it happened in a minority of cases, the discriminative ability of the model was lower. Interestingly, high ferritin and low platelets were associated with higher mortality, a finding in line with a previous study on a subgroup of children with MIS‐C that developed macrophage activation syndrome [26].

Overall, the most relevant variables in our models were simple parameters including haemoglobin, CRP, ferritin, and creatinine, laboratory values easily available in most clinical laboratories worldwide, making our model easily applicable. In addition, these parameters have already been linked with worse clinical presentations in MIS‐C [17, 23], Kawasaki disease [27], and other inflammatory conditions like macrophage activation syndrome [28], indirectly validating the value of the model. Clinically, cough was linked with a higher risk of mechanical ventilation, which could be a sign of pulmonary oedema in children with shock complicating MIS‐C.

Mucositis, rash, conjunctival injection, and gastrointestinal symptoms were generally associated with a lower risk of negative outcomes in our cohort. The more severe spectrum of MIS‐C is, indeed, more characterised by shock, hyperinflammation, and abdominal involvement [11, 12, 23, 26]. We also noted that in our model, gastrointestinal symptoms and conjunctival injection had little to no contribution to the prediction of our outcomes, as these symptoms are very frequent in all patients with MIS‐C [2, 6, 29]. Given the expected low variance, we hypothesised they could have provided low discriminative ability for MIS‐C‐related outcomes.

The approach used in this study, through the web application, can be easily tested and eventually validated in other cohorts, even retrospectively. However, it was notable that this can also serve as an inspiring step for the development of a similar approach for Kawasaki disease, aiming to predict, in this case, coronary abnormalities, which is the most feared outcome for families and clinicians. While treatment resistance can be predicted by scoring systems in many parts of Asia, these algorithms have not worked in multiethnic populations [30, 31, 32, 33]. Ideally, a model similar to the one we developed for MIS‐C can help predict the most severe cases of Kawasaki disease that would benefit from early adjunctive combination therapy. In addition, a similar model could be studied prospectively to evaluate if implementing an early prediction no need for a hyphen tool improves the overall outcomes of Kawasaki disease patients, including time to resolution of pre‐specified outcomes and healthcare‐associated costs.

### 4.1 Strengths and Limitations

The main limitation of our study was—keep an eye on your use of tenses that—the models are based on previously collected data. The presence of missing data for certain variables was a limitation, which reflects the difficulty of data collection in clinical settings, particularly during complex pandemic periods (Figure S1 shows the pattern of missingness of each variable, still not altering the model's validity). This should be Figure S1 and see the guidance on the use of brackets. Despite this limitation, we believe that our model still provides valuable information for clinical practice. In addition, as we only included patients from Latin America, we cannot exclude that, in other populations, different parameters could have a different weight in determining specific outcomes. However, the variables that we considered are widely described in all studies as playing a relevant role in the disease. Although the percentage of death in Latin America due to MIS‐C is higher than in other parts of the world [23, 34], the outcome of death was relatively rare, and in fact for this outcome our model had the lowest discriminative capabilities. Last, *while we have implemented the application, future studies will need to evaluate its usability or clinician feedback*. Nevertheless, a strength is that our study was the largest dataset assessed through machine‐learning approaches to develop an easily available web‐app prediction tool for MIS‐C, paving the way for the implementation of such tools in similar conditions.

## 5 Conclusion

This study developed and validated four machine learning models based on early, easily obtainable clinical parameters for the prediction of multiple adverse outcomes in patients with MIS‐C and made them available for routine use through a web‐based real‐time application. This model can help the clinician risk stratify MIS‐C patients, providing personalised care that could improve patient outcomes.

## Conflicts of Interest

The authors declare no conflicts of interest.

## Supporting information


**Appendix S1:** apa70290‐sup‐0001‐AppendixS1.docx.

## Data Availability

The datasets used and/or analysed during the current study are not publicly available but may be available to qualified researchers on reasonable request from the corresponding author. *Code availability*: The underlying code for this study and training/validation datasets is not publicly available but may be made available to qualified researchers on reasonable request from the corresponding author.
