# Supplementary material for: Development and Validation of Multivariable Machine‐Learning Models for the Prediction of Multisystemic Inflammatory Syndrome Outcomes in Latin American Children
Source: Acta Paediatr. 2025 Sep 1;115(1):133–45. doi: 10.1111/apa.70290 (PMC12687682; doi:10.1111/apa.70290)
Supplement: Supplementary file 1 — Appendix S1: apa70290‐sup‐0001‐AppendixS1.docx. [file APA-115-133-s001.docx]

**Supplementary Material**

**Title: Development and validation of multivariable machine-learning models for the prediction of Multisystemic Inflammatory Syndrome (MIS-C) outcomes in Latin American children: a multicenter study from the multinational REKAMLATINA research network.**

**Authors**

**Summary**

[**Supplementary Table 1. Univariate analysis in the train set.** 2](#_Toc185961542)

[**Supplementary Fig. 1. Missingness details on the full cohort.** 1](#_Toc185961543)

[**Supplementary Fig. 3. PICU admission: model evaluation and variable importance.** 2](#_Toc185961544)

[**Supplementary Fig. 4. PICU admission: model comparison.** 3](#_Toc185961545)

[**Supplementary Fig. 5. Inotropes use: model evaluation and variable importance.** 4](#_Toc185961546)

[**Supplementary Fig. 6. Inotropes use: model comparison.** 5](#_Toc185961547)

[**Supplementary Fig. 7. Mechanic ventilation: model evaluation and variable importance.** 6](#_Toc185961548)

[**Supplementary Fig. 8. Mechanic ventilation: model comparison.** 7](#_Toc185961549)

[**Supplementary Fig. 9. Death: model evaluation and variable importance.** 8](#_Toc185961550)

[**Supplementary Fig. 10. Death: model comparison.** 9](#_Toc185961551)

[**Supplementary Table 2. TRIPOD-AI Checklist.** 10](#_Toc185961552)

Supplementary Table 3. Sensitivity analyses

REKAMLATINA INVESTIGATORS

# **Supplementary Table 1. Univariate analysis in the train set.**

**[A] PICU Admission**

|  | **Missing** | **No** | **Yes** | **P-Value*** |
| --- | --- | --- | --- | --- |
| n |  | 477 (52.3) | 435 (47.7) |  |
| Gender |  |  |  | 0.252 |
| Female |  | 209 (43.8) | 208 (47.8) |  |
| Male |  | 268 (56.2) | 227 (52.2) |  |
| Age | 0 | 5.3 [2.5 - 9.0] | 8.1 [4.0 - 11.8] | <0.001 |
| Fever days | 14 | 5.0 [4.0 - 7.0] | 5.0 [3.0 - 7.0] | 0.008 |
| Dehydration | **9** |  |  | <0.001 |
| No |  | 333 (69.8) | 232 (53.3) |  |
| Yes |  | 141 (29.6) | 197 (45.3) |  |
| Cough | 3 |  |  | 0.003 |
| No |  | 348 (73.0) | 273 (62.8) |  |
| Yes |  | 127 (26.6) | 161 (37.0) |  |
| Conjunctivitis | 3 |  |  | 0.007 |
| No |  | 159 (33.3) | 180 (41.4) |  |
| Yes |  | 318 (66.7) | 252 (57.9) |  |
| Mucositis | 4 |  |  | <0.001 |
| No |  | 230 (48.2) | 278 (63.9) |  |
| Yes |  | 246 (51.6) | 154 (35.4) |  |
| Lymphadenopathy | 7 |  |  | <0.001 |
| No |  | 317 (66.5) | 340 (78.2) |  |
| Yes |  | 156 (32.7) | 92 (21.1) |  |
| Edema extremitis | 6 |  |  | 0.006 |
| No |  | 262 (54.9) | 274 (63.0) |  |
| Yes |  | 214 (44.9) | 156 (35.9) |  |
| Rash | 2 |  |  | 0.001 |
| No |  | 166 (34.8) | 198 (45.5) |  |
| Yes |  | 311 (65.2) | 235 (54.0) |  |
| Convulsions | 8 |  |  | 0.002 |
| No |  | 465 (97.5) | 403 (92.6) |  |
| Yes |  | 9 (1.9) | 27 (6.2) |  |
| Shock | 3 |  |  | <0.001 |
| No |  | 398 (83.4) | 155 (35.6) |  |
| Yes |  | 77 (16.1) | 279 (64.1) |  |
| Abdominal pain | 12 |  |  | 0.201 |
| No |  | 176 (36.9) | 136 (31.3) |  |
| Yes |  | 295 (61.8) | 293 (67.4) |  |
| Vomiting |  |  |  | 0.054 |
| No |  | 222 (46.5) | 174 (40.0) |  |
| Yes |  | 255 (53.5) | 261 (60.0) |  |
| Diarrhea | 2 |  |  | 0.276 |
| No |  | 272 (57.0) | 225 (51.7) |  |
| Yes |  | 204 (42.8) | 209 (48.0) |  |
| Haemoglobin | 0 | 11.4 [10.4 - 12.4] | 10.9 [9.4 - 12.3] | <0.001 |
| Platelets | 4 | 231.0 [144.2 - 334.8] | 147.0 [85.0 - 234.8] | <0.001 |
| Lymphocites | 17 | 1500.0 [834.8 - 2589.8] | 951.0 [540.0 - 1767.0] | <0.001 |
| CRP | 40 | 11.0 [5.0 - 22.4] | 17.5 [9.0 - 26.9] | <0.001 |
| PCT | 458 | 1.8 [0.4 - 6.3] | 6.7 [1.8 - 21.7] | <0.001 |
| Creatinine | 28 | 0.4 [0.3 - 0.6] | 0.6 [0.4 - 0.8] | <0.001 |
| ALT | 22 | 35.0 [22.0 - 72.0] | 47.5 [26.0 - 84.0] | 0.001 |
| AST | 20 | 40.0 [27.0 - 66.0] | 47.0 [30.0 - 90.0] | 0.001 |
| Ferritin | 154 | 326.0 [162.2 - 674.1] | 563.2 [303.5 - 1210.5] | <0.001 |
| pro-BNP | 489 | 650.5 [201.3 - 2332.8] | 3197.0 [568.0 - 9935.5] | <0.001 |
| Echocardiography normal | 69 |  |  | <0.001 |
| No |  | 178 (37.3) | 240 (55.2) |  |
| Yes |  | 257 (53.9) | 168 (38.6) |  |
| ECG normal | 346 |  |  | 0.016 |
| No |  | 62 (13.0) | 79 (18.2) |  |
| Yes |  | 242 (50.7) | 183 (42.1) |  |

Continous varibales are reported as median [IQR]. Categorical variables as counts (percentage). *Mann-Whitney U-test for continuous variable s and Chi-Square test (or Fisher test) for categorical variables.

**[B] Inotropes use**

|  |  | Missing | No | Yes | P-Value |
| --- | --- | --- | --- | --- | --- |
|  |  |  |  |  |  |
| n |  |  | 535 | 377 |  |
| Gender | **Female** |  | 235 (43.9) | 182 (48.3) | 0.218 |
|  | **Male** |  | 300 (56.1) | 195 (51.7) |  |
| Age |  | 0 | 5.3 [2.4 - 9.0] | 8.3 [4.2 - 12.0] | <0.001 |
| Fever days |  | 14 | 5.0 [4.0 - 7.0] | 5.0 [3.0 - 7.0] | 0.091 |
| Dehydration | **No** |  | 364 (68.0) | 201 (53.3) | <0.001 |
|  | **Missing** |  | 4 (0.7) | 5 (1.3) |  |
|  | **Yes** |  | 167 (31.2) | 171 (45.4) |  |
| Cough | **No** |  | 382 (71.4) | 239 (63.4) | 0.034 |
|  | **Missing** |  | 2 (0.4) | 1 (0.3) |  |
|  | **Yes** |  | 151 (28.2) | 137 (36.3) |  |
| Conjunctivitis | **No** |  | 187 (35.0) | 152 (40.3) | 0.250 |
|  | **Missing** |  | 2 (0.4) | 1 (0.3) |  |
|  | **Yes** |  | 346 (64.7) | 224 (59.4) |  |
| Mucositis | **No** |  | 275 (51.4) | 233 (61.8) | 0.002 |
|  | **Missing** |  | 1 (0.2) | 3 (0.8) |  |
|  | **Yes** |  | 259 (48.4) | 141 (37.4) |  |
| Lymphadenopathy | **No** |  | 371 (69.3) | 286 (75.9) | 0.090 |
|  | **Missing** |  | 4 (0.7) | 3 (0.8) |  |
|  | **Yes** |  | 160 (29.9) | 88 (23.3) |  |
| Edema extremitis | **No** |  | 302 (56.4) | 234 (62.1) | 0.084 |
|  | **Missing** |  | 2 (0.4) | 4 (1.1) |  |
|  | **Yes** |  | 231 (43.2) | 139 (36.9) |  |
| Rash | **No** |  | 191 (35.7) | 173 (45.9) | 0.002 |
|  | **Yes** |  | 344 (64.3) | 202 (53.6) |  |
|  | **Missing** |  |  | 2 (0.5) |  |
| Convulsions | **No** |  | 526 (98.3) | 342 (90.7) | <0.001 |
|  | **Missing** |  | 3 (0.6) | 5 (1.3) |  |
|  | **Yes** |  | 6 (1.1) | 30 (8.0) |  |
| Shock | **No** |  | 452 (84.5) | 101 (26.8) | <0.001 |
|  | **Missing** |  | 2 (0.4) | 1 (0.3) |  |
|  | **Yes** |  | 81 (15.1) | 275 (72.9) |  |
| Belly pain | **No** |  | 199 (37.2) | 113 (30.0) | 0.070 |
|  | **Missing** |  | 6 (1.1) | 6 (1.6) |  |
|  | **Yes** |  | 330 (61.7) | 258 (68.4) |  |
| Vomiting | **No** |  | 250 (46.7) | 146 (38.7) | 0.020 |
|  | **Yes** |  | 285 (53.3) | 231 (61.3) |  |
| Diarrhea | **No** |  | 298 (55.7) | 199 (52.8) | 0.670 |
|  | **Missing** |  | 1 (0.2) | 1 (0.3) |  |
|  | **Yes** |  | 236 (44.1) | 177 (46.9) |  |
| Haemoglobin |  | 0 | 11.4 [10.2 - 12.4] | 10.9 [9.4 - 12.3] | <0.001 |
| Platelets |  | 4 | 219.0 [142.0 - 331.2] | 144.5 [80.6 - 228.1] | <0.001 |
| Lymphocites |  | 17 | 1500.0 [809.0 - 2600.0] | 920.0 [537.5 - 1700.0] | <0.001 |
| CRP |  | 40 | 11.0 [5.1 - 22.3] | 18.7 [9.6 - 27.5] | <0.001 |
| PCT |  | 458 | 1.8 [0.4 - 6.3] | 7.5 [2.3 - 23.7] | <0.001 |
| Creatinine |  | 28 | 0.4 [0.3 - 0.6] | 0.6 [0.4 - 0.9] | <0.001 |
| ALT |  | 22 | 35.0 [21.9 - 71.8] | 49.0 [28.0 - 87.0] | <0.001 |
| AST |  | 20 | 41.0 [27.0 - 67.0] | 49.0 [30.0 - 92.0] | 0.001 |
| Ferritin |  | 154 | 303.5 [163.2 - 619.8] | 653.0 [379.5 - 1246.4] | <0.001 |
| pro-BNP |  | 489 | 642.5 [200.0 - 2278.5] | 3439.0 [817.0 - 11000.0] | <0.001 |
| Echocardiography normal | **No** |  | 202 (37.8) | 216 (57.3) | <0.001 |
|  | **Missing** |  | 43 (8.0) | 26 (6.9) |  |
|  | **Yes** |  | 290 (54.2) | 135 (35.8) |  |
| ECG normal | **No** |  | 70 (13.1) | 71 (18.8) | 0.008 |
|  | **Missing** |  | 195 (36.4) | 151 (40.1) |  |
|  | **Yes** |  | 270 (50.5) | 155 (41.1) |  |

**[C] Mechanic ventilation**

|  |  | Missing | No | Yes | P-Value |
| --- | --- | --- | --- | --- | --- |
|  |  |  |  |  |  |
| n |  |  | 722 | 190 |  |
| Gender | **Female** |  | 312 (43.2) | 105 (55.3) | 0.004 |
|  | **Male** |  | 410 (56.8) | 85 (44.7) |  |
| Age |  | 0 | 6.5 [3.0 - 10.0] | 8.1 [4.0 - 11.6] | 0.005 |
| Fever days |  | 14 | 5.0 [4.0 - 7.0] | 5.0 [3.0 - 6.8] | <0.001 |
| Dehydration | **No** |  | 456 (63.2) | 109 (57.4) | 0.342 |
|  | **Missing** |  | 7 (1.0) | 2 (1.1) |  |
|  | **Yes** |  | 259 (35.9) | 79 (41.6) |  |
| Cough | **No** |  | 520 (72.0) | 101 (53.2) | <0.001 |
|  | **Missing** |  | 2 (0.3) | 1 (0.5) |  |
|  | **Yes** |  | 200 (27.7) | 88 (46.3) |  |
| Conjunctivitis | **No** |  | 245 (33.9) | 94 (49.5) | <0.001 |
|  | **Missing** |  | 2 (0.3) | 1 (0.5) |  |
|  | **Yes** |  | 475 (65.8) | 95 (50.0) |  |
| Mucositis | **No** |  | 375 (51.9) | 133 (70.0) | <0.001 |
|  | **Missing** |  | 2 (0.3) | 2 (1.1) |  |
|  | **Yes** |  | 345 (47.8) | 55 (28.9) |  |
| Lymphadenopathy | **No** |  | 509 (70.5) | 148 (77.9) | 0.028 |
|  | **Missing** |  | 4 (0.6) | 3 (1.6) |  |
|  | **Yes** |  | 209 (28.9) | 39 (20.5) |  |
| Edema extremitis | **No** |  | 403 (55.8) | 133 (70.0) | 0.002 |
|  | **Missing** |  | 5 (0.7) | 1 (0.5) |  |
|  | **Yes** |  | 314 (43.5) | 56 (29.5) |  |
| Rash | **No** |  | 269 (37.3) | 95 (50.0) | <0.001 |
|  | **Yes** |  | 453 (62.7) | 93 (48.9) |  |
|  | **Missing** |  |  | 2 (1.1) |  |
| Convulsions | **No** |  | 702 (97.2) | 166 (87.4) | <0.001 |
|  | **Missing** |  | 5 (0.7) | 3 (1.6) |  |
|  | **Yes** |  | 15 (2.1) | 21 (11.1) |  |
| Shock | **No** |  | 512 (70.9) | 41 (21.6) | <0.001 |
|  | **Missing** |  | 2 (0.3) | 1 (0.5) |  |
|  | **Yes** |  | 208 (28.8) | 148 (77.9) |  |
| Belly pain | **No** |  | 247 (34.2) | 65 (34.2) | 0.560 |
|  | **Missing** |  | 8 (1.1) | 4 (2.1) |  |
|  | **Yes** |  | 467 (64.7) | 121 (63.7) |  |
| Vomiting | **No** |  | 316 (43.8) | 80 (42.1) | 0.742 |
|  | **Yes** |  | 406 (56.2) | 110 (57.9) |  |
| Diarrhea | **No** |  | 385 (53.3) | 112 (58.9) | 0.309 |
|  | **Missing** |  | 2 (0.3) |  |  |
|  | **Yes** |  | 335 (46.4) | 78 (41.1) |  |
| Haemoglobin |  | 0 | 11.3 [10.1 - 12.4] | 10.6 [9.0 - 12.2] | <0.001 |
| Platelets |  | 4 | 206.0 [126.5 - 301.5] | 123.0 [67.0 - 200.0] | <0.001 |
| Lymphocites |  | 17 | 1320.0 [724.0 - 2368.0] | 888.5 [562.2 - 1731.8] | <0.001 |
| CRP |  | 40 | 13.8 [6.4 - 23.9] | 18.1 [9.0 - 27.5] | 0.003 |
| PCT |  | 458 | 2.4 [0.7 - 8.9] | 8.9 [2.0 - 24.7] | <0.001 |
| Creatinine |  | 28 | 0.5 [0.3 - 0.6] | 0.7 [0.4 - 1.0] | <0.001 |
| ALT |  | 22 | 36.0 [22.0 - 70.5] | 59.0 [33.0 - 109.0] | <0.001 |
| AST |  | 20 | 41.6 [27.0 - 67.0] | 61.0 [36.0 - 126.8] | <0.001 |
| Ferritin |  | 154 | 357.9 [185.6 - 720.0] | 838.0 [479.8 - 1822.5] | <0.001 |
| pro-BNP |  | 489 | 903.0 [228.3 - 3847.5] | 3500.7 [781.5 - 18317.0] | <0.001 |
| Echocardiography normal | **No** |  | 314 (43.5) | 104 (54.7) | <0.001 |
|  | **Missing** |  | 47 (6.5) | 22 (11.6) |  |
|  | **Yes** |  | 361 (50.0) | 64 (33.7) |  |
| ECG normal | **No** |  | 100 (13.9) | 41 (21.6) | 0.003 |
|  | **Missing** |  | 267 (37.0) | 79 (41.6) |  |
|  | **Yes** |  | 355 (49.2) | 70 (36.8) |  |

**[D] Death**

|  |  | Missing | No | Yes | P-Value |
| --- | --- | --- | --- | --- | --- |
|  |  |  |  |  |  |
| n |  |  | 863 | 49 |  |
| Gender | **Female** |  | 392 (45.4) | 25 (51.0) | 0.537 |
|  | **Male** |  | 471 (54.6) | 24 (49.0) |  |
| Age |  | 0 | 6.7 [3.0 - 10.3] | 8.2 [3.1 - 12.0] | 0.218 |
| Fever days |  | 14 | 5.0 [4.0 - 7.0] | 4.0 [3.0 - 5.0] | 0.006 |
| Dehydration | **No** |  | 541 (62.7) | 24 (49.0) | 0.137 |
|  | **Missing** |  | 8 (0.9) | 1 (2.0) |  |
|  | **Yes** |  | 314 (36.4) | 24 (49.0) |  |
| Cough | **No** |  | 598 (69.3) | 23 (46.9) | 0.001 |
|  | **Missing** |  | 2 (0.2) | 1 (2.0) |  |
|  | **Yes** |  | 263 (30.5) | 25 (51.0) |  |
| Conjunctivitis | **No** |  | 308 (35.7) | 31 (63.3) | <0.001 |
|  | **Missing** |  | 2 (0.2) | 1 (2.0) |  |
|  | **Yes** |  | 553 (64.1) | 17 (34.7) |  |
| Mucositis | **No** |  | 470 (54.5) | 38 (77.6) | 0.006 |
|  | **Missing** |  | 4 (0.5) |  |  |
|  | **Yes** |  | 389 (45.1) | 11 (22.4) |  |
| Lymphadenopathy | **No** |  | 618 (71.6) | 39 (79.6) | 0.225 |
|  | **Missing** |  | 6 (0.7) | 1 (2.0) |  |
|  | **Yes** |  | 239 (27.7) | 9 (18.4) |  |
| Edema extremitis | **No** |  | 501 (58.1) | 35 (71.4) | 0.166 |
|  | **Missing** |  | 6 (0.7) |  |  |
|  | **Yes** |  | 356 (41.3) | 14 (28.6) |  |
| Rash | **No** |  | 330 (38.2) | 34 (69.4) | <0.001 |
|  | **Missing** |  | 1 (0.1) | 1 (2.0) |  |
|  | **Yes** |  | 532 (61.6) | 14 (28.6) |  |
| Convulsions | **No** |  | 830 (96.2) | 38 (77.6) | <0.001 |
|  | **Missing** |  | 6 (0.7) | 2 (4.1) |  |
|  | **Yes** |  | 27 (3.1) | 9 (18.4) |  |
| Shock | **No** |  | 538 (62.3) | 15 (30.6) | <0.001 |
|  | **Missing** |  | 3 (0.3) |  |  |
|  | **Yes** |  | 322 (37.3) | 34 (69.4) |  |
| Belly pain | **No** |  | 288 (33.4) | 24 (49.0) | 0.066 |
|  | **Missing** |  | 11 (1.3) | 1 (2.0) |  |
|  | **Yes** |  | 564 (65.4) | 24 (49.0) |  |
| Vomiting | **No** |  | 372 (43.1) | 24 (49.0) | 0.510 |
|  | **Yes** |  | 491 (56.9) | 25 (51.0) |  |
| Diarrhea | **No** |  | 463 (53.7) | 34 (69.4) | 0.096 |
|  | **Missing** |  | 2 (0.2) |  |  |
|  | **Yes** |  | 398 (46.1) | 15 (30.6) |  |
| Haemoglobin |  | 0 | 11.2 [10.0 - 12.3] | 10.7 [9.3 - 12.2] | 0.096 |
| Platelets |  | 4 | 189.0 [112.0 - 290.0] | 114.5 [52.8 - 212.6] | <0.001 |
| Lymphocites |  | 17 | 1200.0 [647.5 - 2262.0] | 1200.0 [467.0 - 2595.0] | 0.877 |
| CRP |  | 40 | 14.7 [6.7 - 24.8] | 12.8 [6.2 - 28.3] | 0.894 |
| PCT |  | 458 | 3.0 [0.8 - 12.7] | 7.0 [2.9 - 17.1] | 0.155 |
| Creatinine |  | 28 | 0.5 [0.3 - 0.7] | 1.0 [0.4 - 1.7] | <0.001 |
| ALT |  | 22 | 39.0 [23.0 - 74.0] | 85.5 [41.8 - 149.0] | <0.001 |
| AST |  | 20 | 43.0 [28.0 - 76.0] | 68.0 [35.5 - 256.0] | <0.001 |
| Ferritin |  | 154 | 416.5 [214.1 - 839.0] | 1695.5 [765.5 - 3858.0] | <0.001 |
| pro-BNP |  | 489 | 1222.0 [279.5 - 5264.5] | 3197.5 [522.0 - 5575.8] | 0.245 |
| Echocardiography normal | **No** |  | 394 (45.7) | 24 (49.0) | <0.001 |
|  | **Missing** |  | 55 (6.4) | 14 (28.6) |  |
|  | **Yes** |  | 414 (48.0) | 11 (22.4) |  |
| ECG normal | **No** |  | 126 (14.6) | 15 (30.6) | <0.001 |
|  | **Missing** |  | 321 (37.2) | 25 (51.0) |  |
|  | **Yes** |  | 416 (48.2) | 9 (18.4) |  |

Continous varibales are reported as median [IQR]. Categorical variables as counts (percentage). *Mann-Whitney U-test for continuous variable s and Chi-Square test (or Fisher test) for categorical variables.

# **Supplementary Fig. 1. Missingness details on the full cohort.**

**
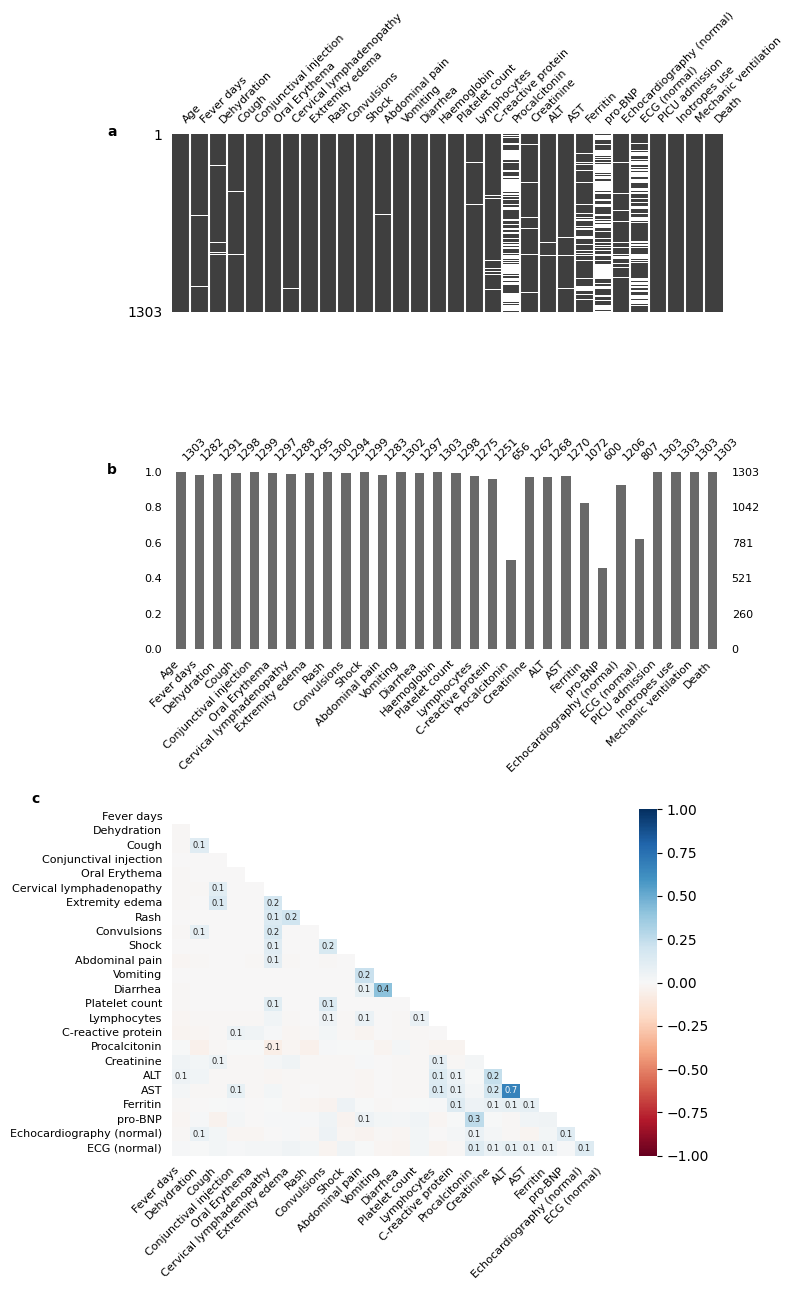
**

**Details for missing values. a.** Missingness map. **b**. Missingness barplot. **c.** Missingness correlation heatmap.

# **Supplementary Fig. 2. PICU admission: model evaluation and variable importance.**


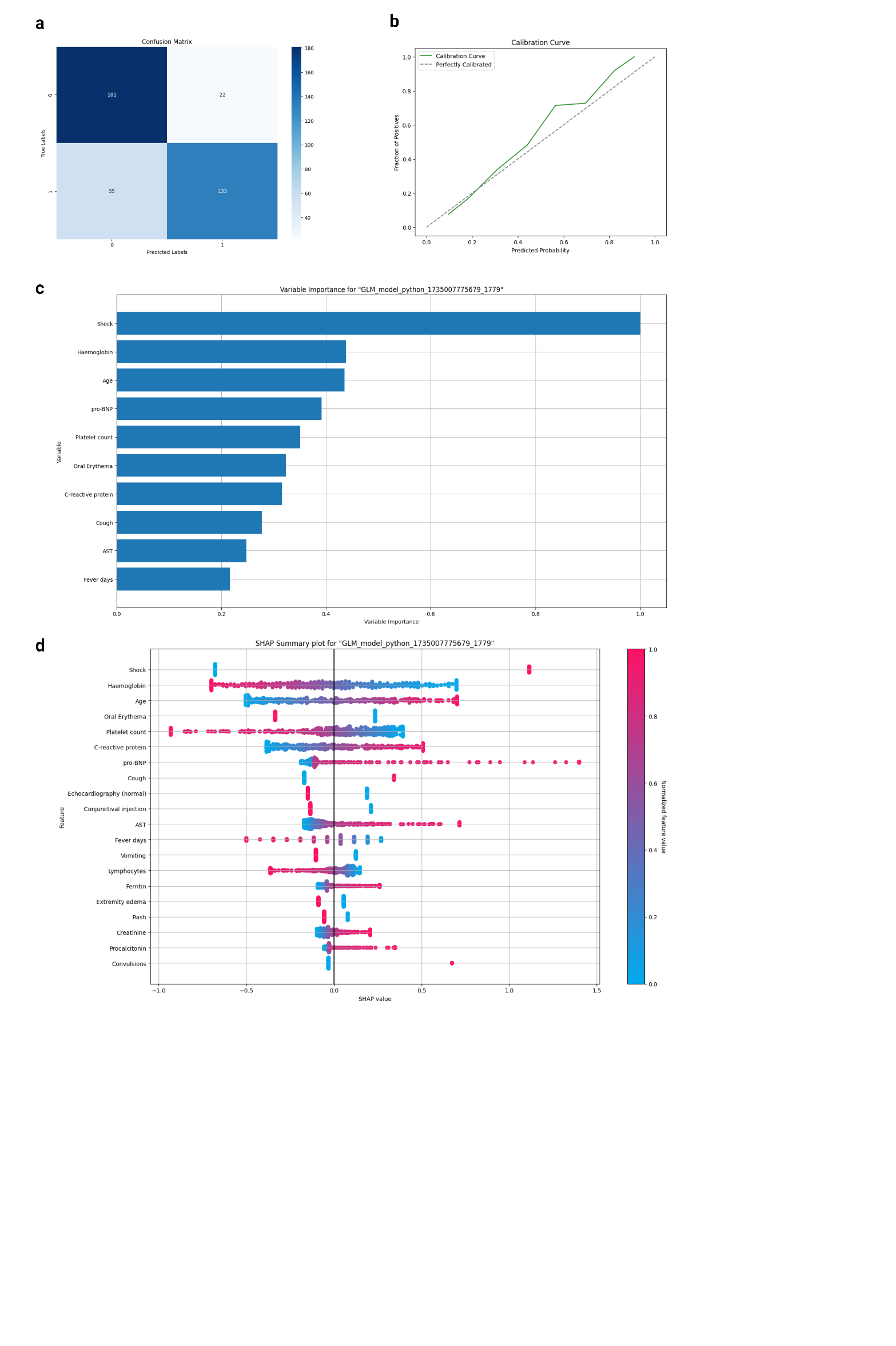


**Model evaluation and variable importance for PICU admission. a.** Confusion Matrix for the random forest model (0=No PICU, 1=PICU). **b.** Calibration curve for the random forest model. **c.** Variable importance for the GLM (Elastic Net). **d.** SHAP values for the GLM (Elastic Net). SHAP values for the random forest model are reported in the main text.

# **Supplementary Fig. 3. PICU admission: model comparison.**

**
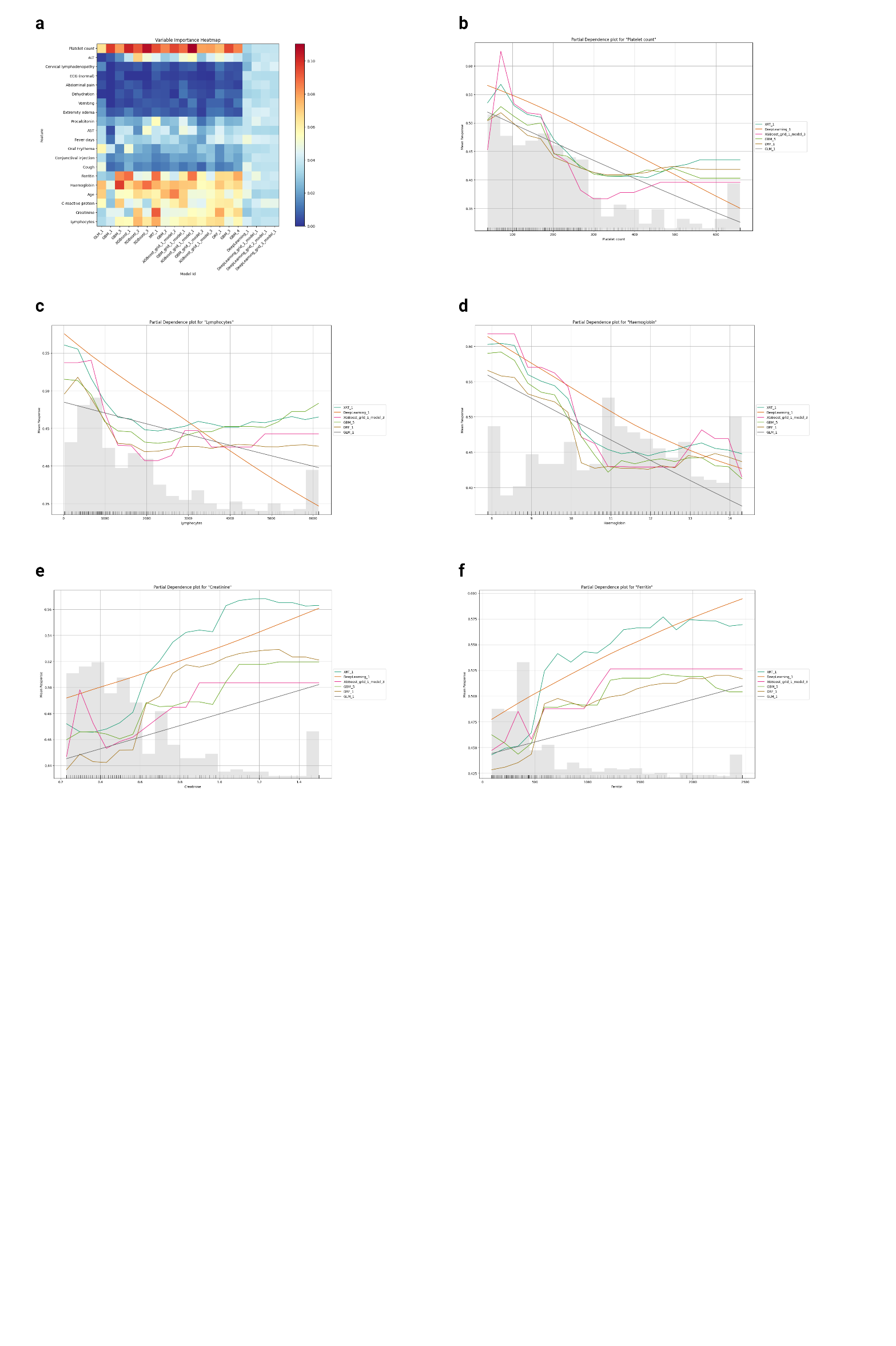
**

**Model comparison for PICU admission. a.** Variable importance heatmap for the different models. higher variable importance for the corresponding model is reported in red, while low importance in blue. **b-f** Partial dependence plots. Each line represents a different model. **b**: platelets; **c**: lymphocytes; **d**: haemoglobin; **e:** creatinine; **f**: ferritin.

# **Supplementary Fig. 4. Inotropes use: model evaluation and variable importance.**


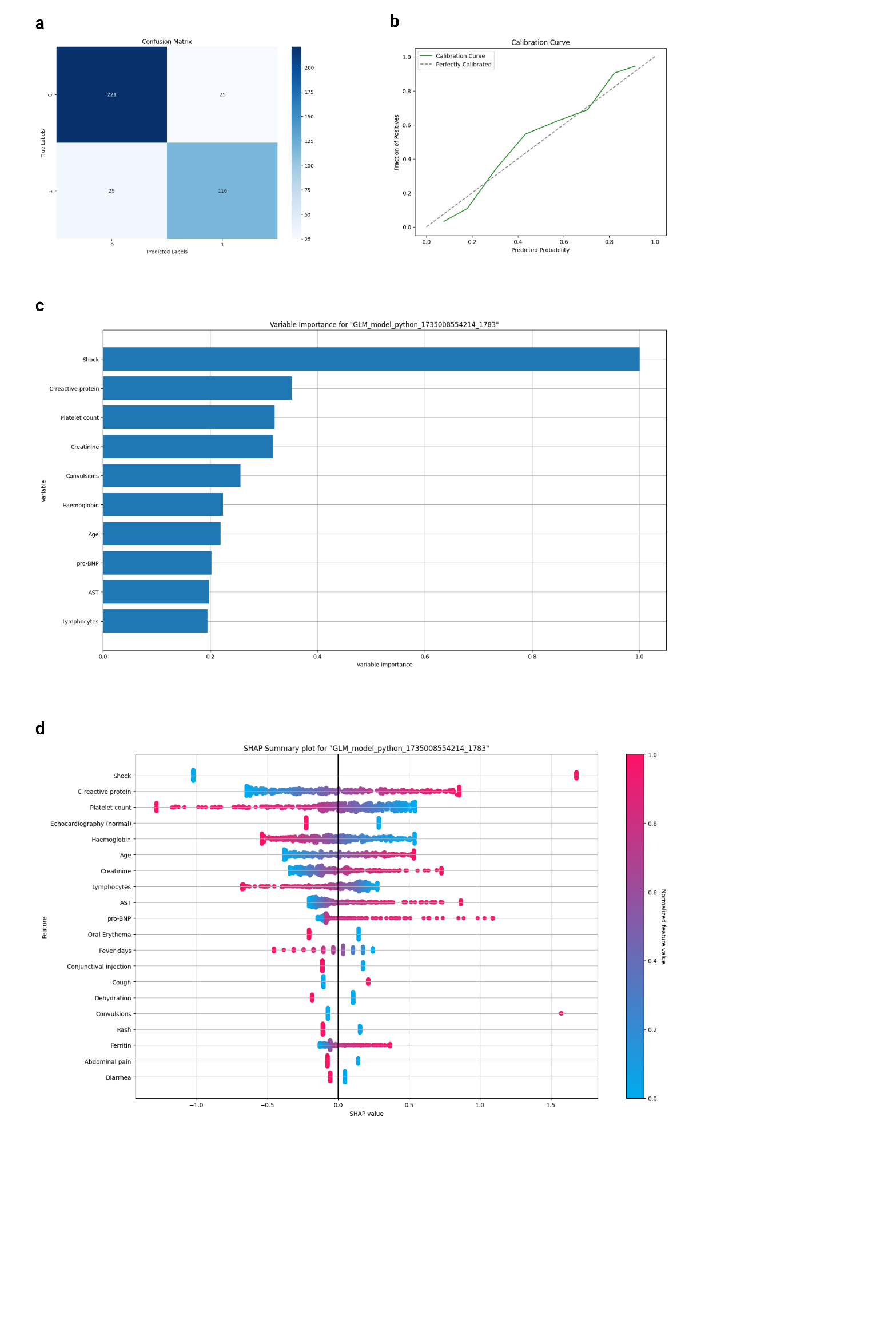


**Model evaluation and variable importance for inotropes use. a.** Confusion Matrix for the random forest model (0=No inotropes, 1=inotropes). **b.** Calibration curve for the random forest model. **c.** Variable importance for the GLM (Elastic Net). **d.** SHAP values for the GLM (Elastic Net). SHAP values for the random forest model are reported in the main text.

# **Supplementary Fig. 5. Inotropes use: model comparison.**


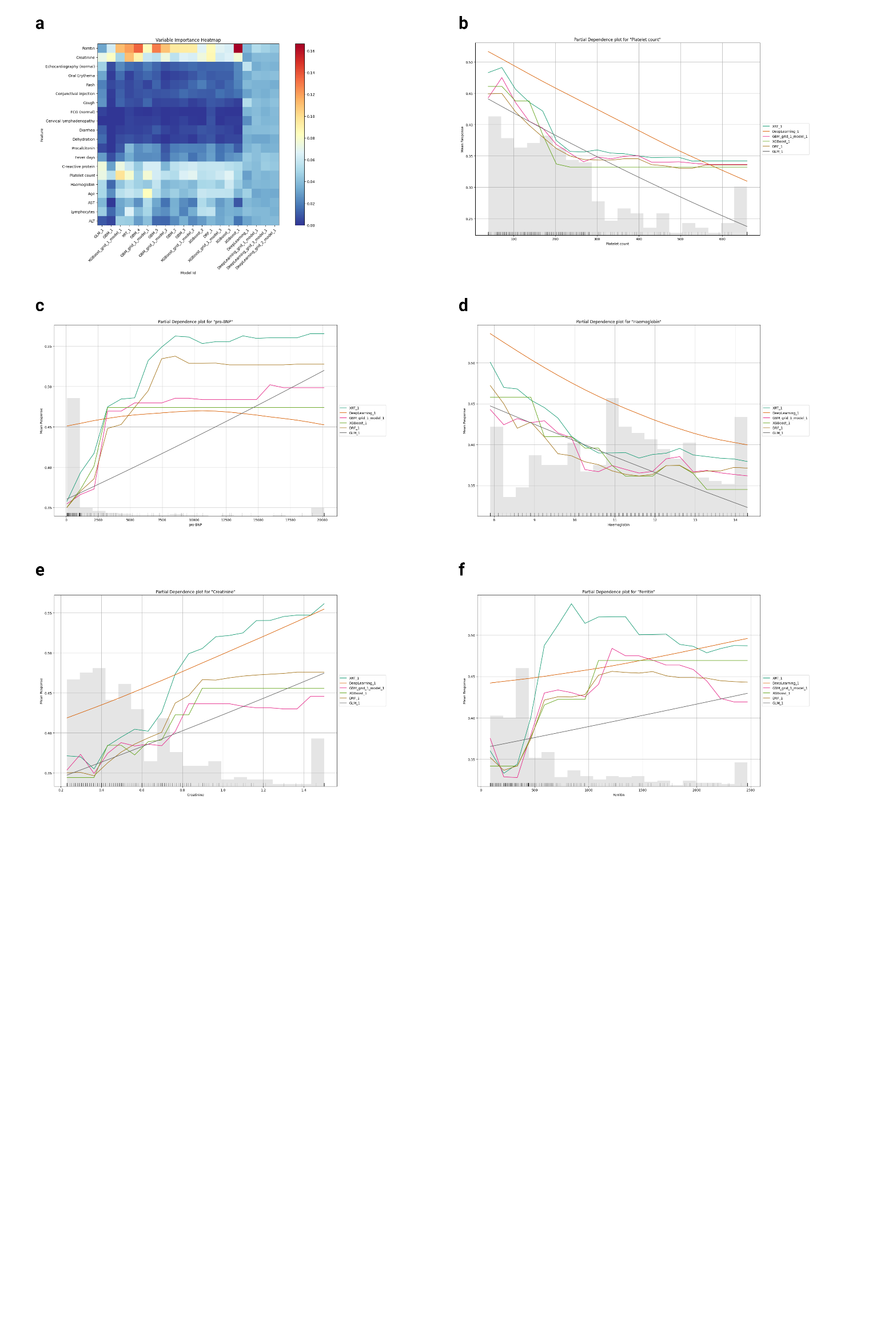


**Model comparison for inotropic use. a.** Variable importance heatmap for the different models. higher variable importance for the corresponding model is reported in red, while low importance in blue. **b-f** Partial dependence plots. Each line represents a different model. **b**: platelets; **c**: pro-BNP; **d**: haemoglobin; **e**: creatinine; **f**: ferritin.

# **Supplementary Fig. 6. Mechanic ventilation: model evaluation and variable importance.**


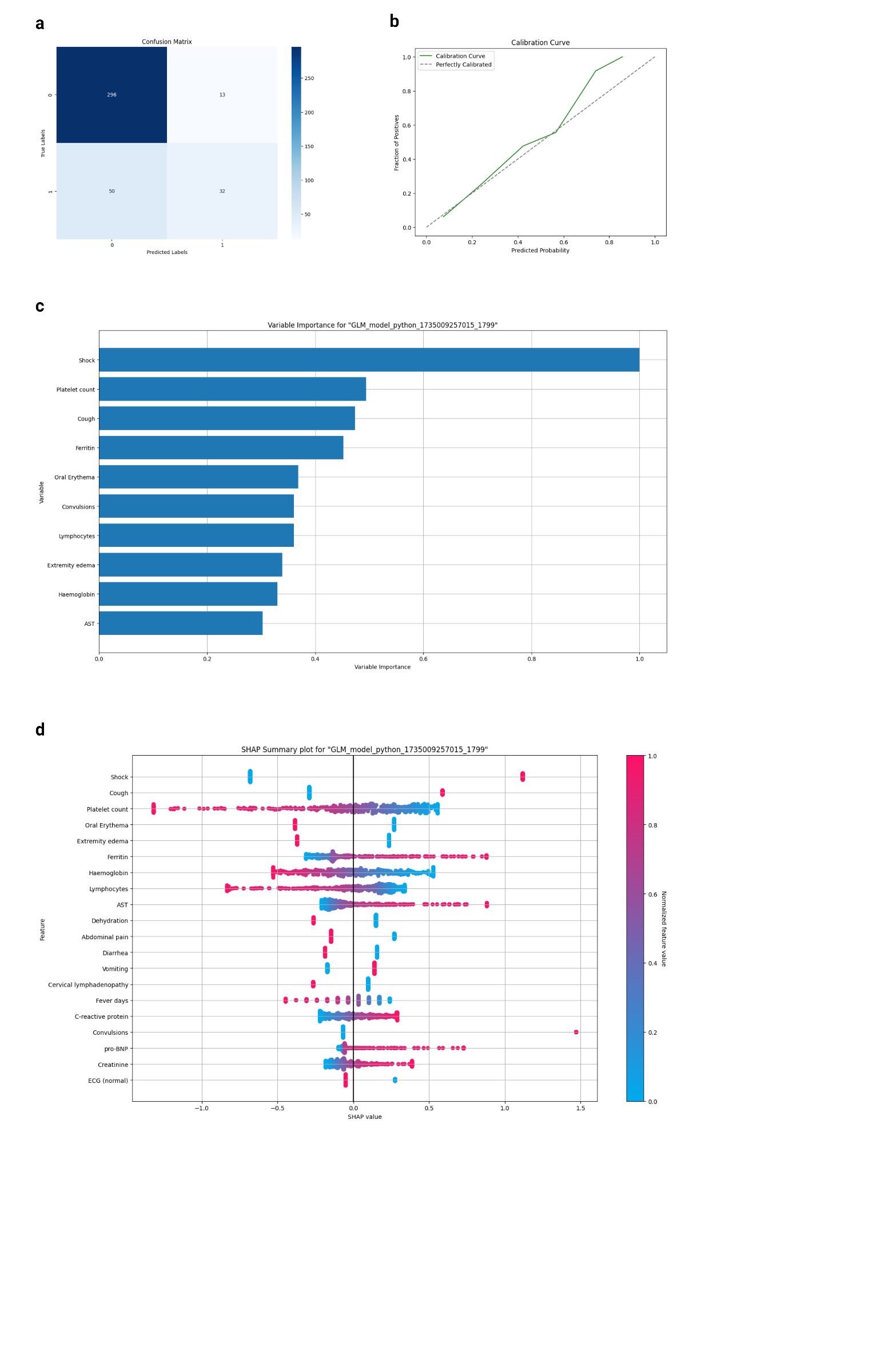


**Model evaluation and variable importance for mechanic ventilation. a.** Confusion Matrix for the random forest model (0=No VM, 1=VM). **b.** Calibration curve for the random forest model. **c.** Variable importance for the GLM (Elastic Net). **d.** SHAP values for the GLM (Elastic Net). SHAP values for the random forest model are reported in the main text.

# **Supplementary Fig. 7. Mechanic ventilation: model comparison.**


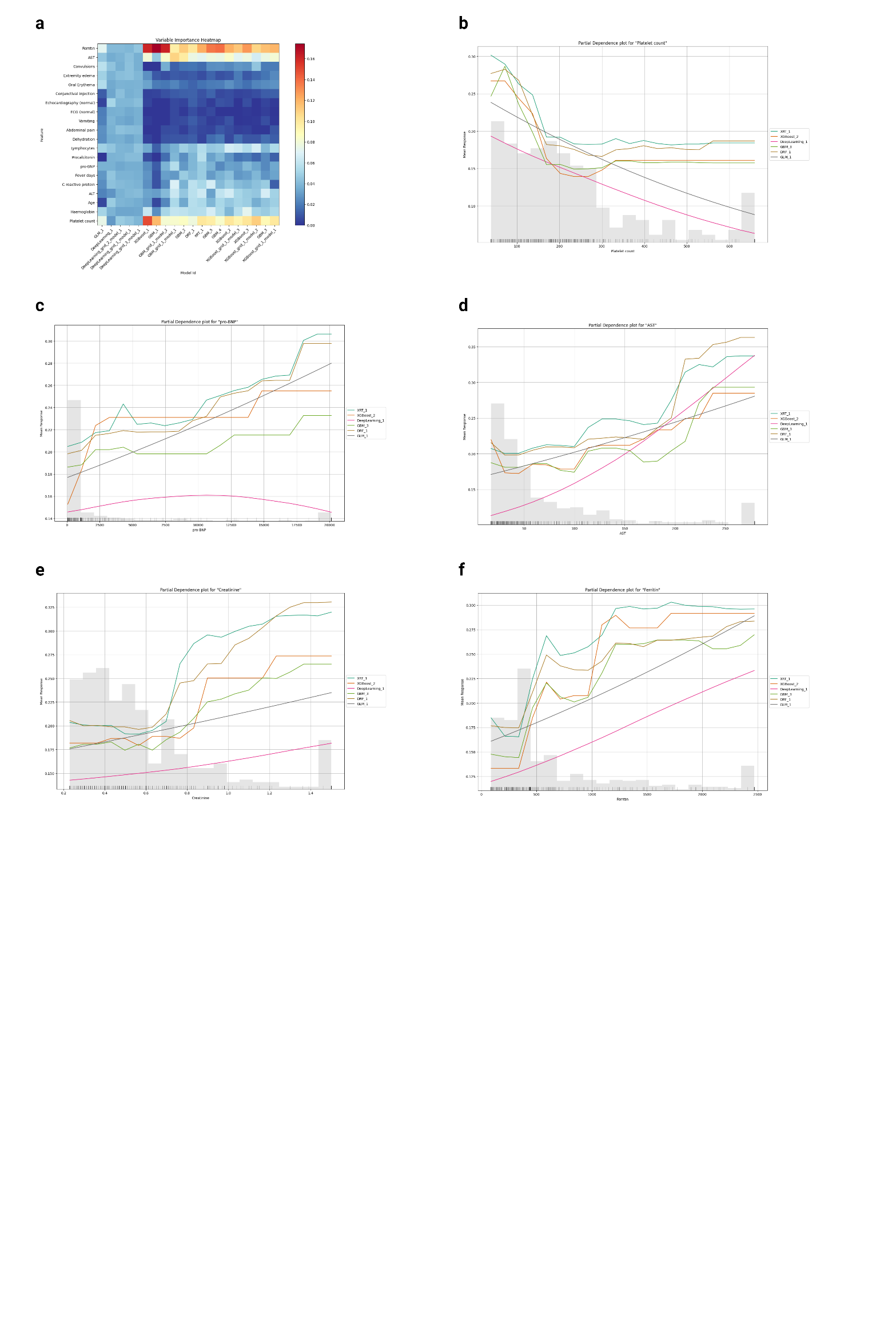


**Model comparison for mechanic ventilation. a.** Variable importance heatmap for the different models. higher variable importance for the corresponding model is reported in red, while low importance in blue. **b-f** Partial dependence plots. Each line represents a different model. **b**: platelets; **c**: pro-BNP; **d**: AST; **e**: creatinine; **f**: ferritin.

# **Supplementary Fig. 8. Death: model evaluation and variable importance.**

**
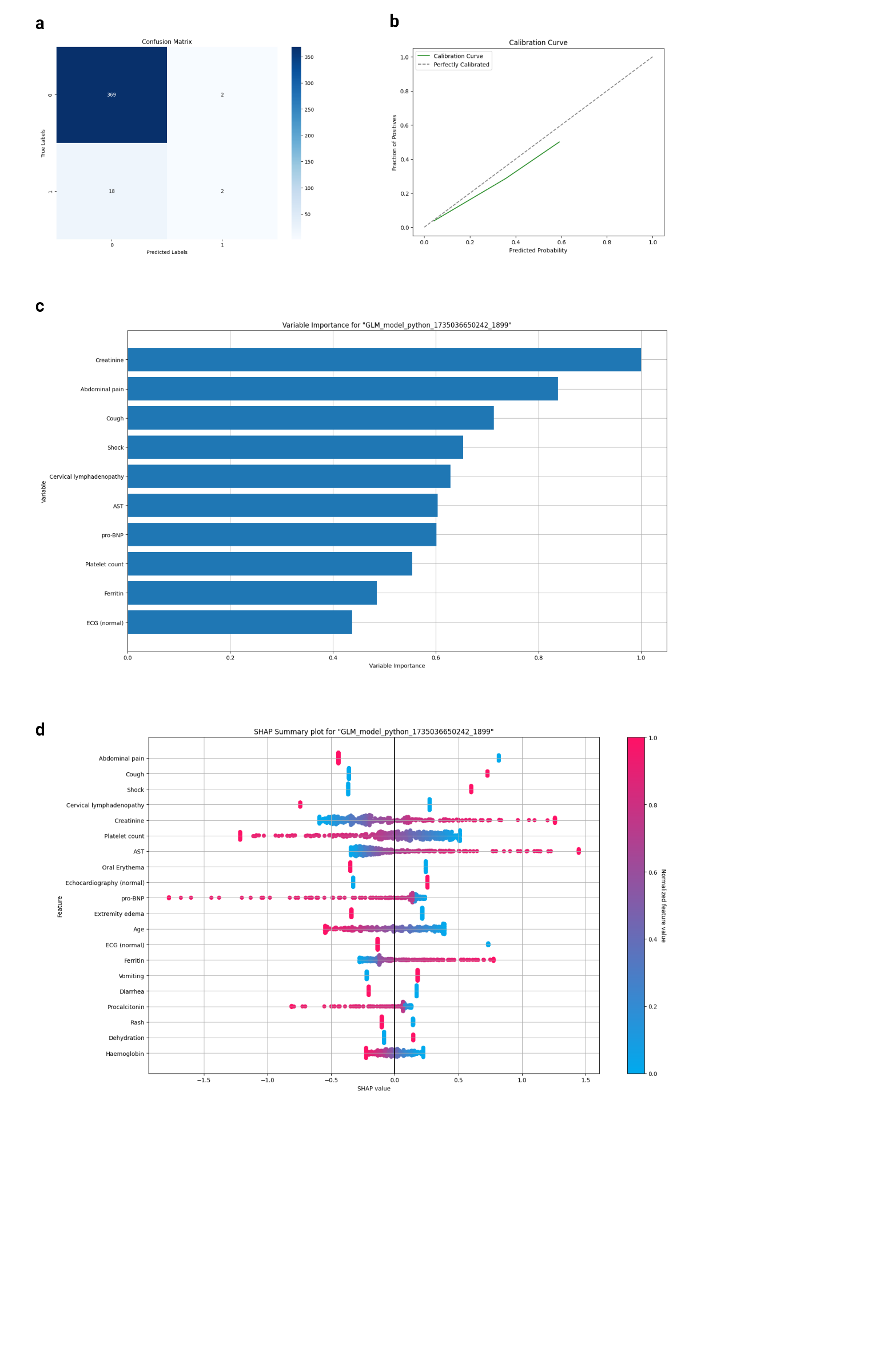
**

**Model evaluation and variable importance for death. a.** Confusion Matrix for the random forest model (0=No death, 1=Death). **b.** Calibration curve for the random forest model. **c.** Variable importance for the GLM (Elastic Net). **d.** SHAP values for the GLM (Elastic Net). SHAP values for the random forest model are reported in the main text.

# **Supplementary Fig. 9. Death: model comparison.**


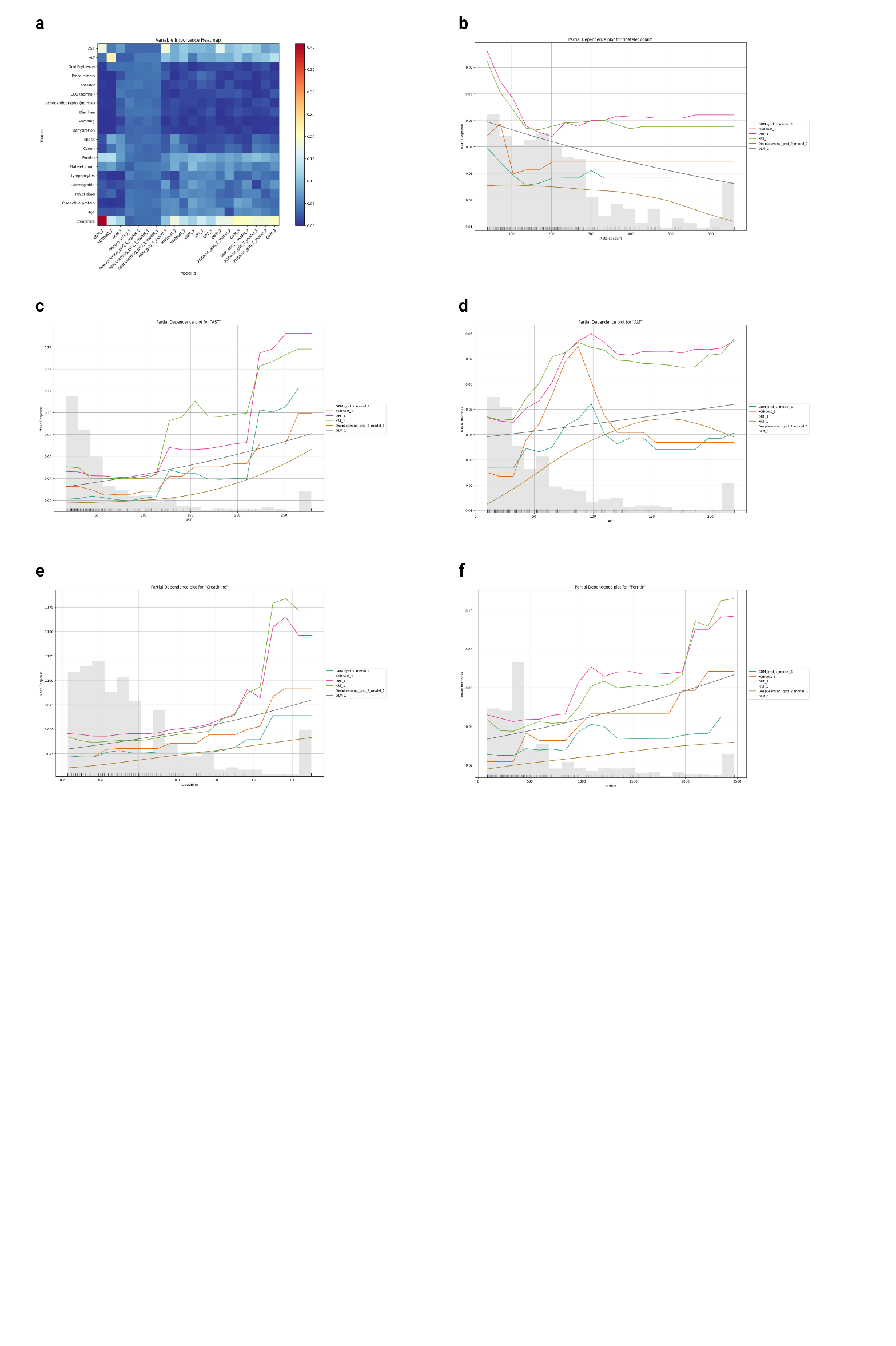


**Model comparison for death. a.** Variable importance heatmap for the different models. higher variable importance for the corresponding model is reported in red, while low importance in blue. **b-o** Partial dependence plots. Each line represents a different model. **b-f** Partial dependence plots. Each line represents a different model. **b**: platelets; **c**: AST; **d**: ALT; **e**: creatinine; **f**: ferritin.

### **Supplementary Table 2. TRIPOD-AI Checklist.**

| **Section/Topic Item Development Checklist item**  **/ evaluation**^1^ | | | | **Reported on page** |
| --- | --- | --- | --- | --- |
| **TITLE** | | | |  |
| *Title* | 1 | D;E | Identify the study as developing or evaluating the performance of a multivariable prediction model, the target population, and the outcome to be predicted | 1 |
| **ABSTRACT** | | | | |
| *Abstract* | 2 | D;E | See TRIPOD+AI for Abstracts checklist | 3-4 |
| **INTRODUCTION** | | | | |
| *Background* | 3a | D;E | Explain the healthcare context (including whether diagnostic or prognostic) and rationale for developing or evaluating the prediction model, including references to existing models | 7-8 |
|  | 3b | D;E | Describe the target population and the intended purpose of the prediction model in the context of the care pathway, including its intended users (e.g., healthcare professionals, patients, public) | 7-8 |
|  | 3c | D;E | Describe any known health inequalities between sociodemographic groups | 7-8 |
| *Objectives* | 4 | D;E | Specify the study objectives, including whether the study describes the development or validation of a prediction model (or both) | 8 |
| **METHODS** | | | | |
| *Data* | 5a | D;E | Describe the sources of data separately for the development and evaluation datasets (e.g., randomised trial, cohort, routine care or registry data), the rationale for using these data, and representativeness of the data | 9 |
|  | 5b | D;E | Specify the dates of the collected participant data, including start and end of participant accrual; and, if applicable, end of follow-up | 9 |
| *Participants* | 6a | D;E | Specify key elements of the study setting (e.g., primary care, secondary care, general population)  including the number and location of centres | 9-10 |
|  | 6b | D;E | Describe the eligibility criteria for study participants | 9-10 |
|  | 6c | D;E | Give details of any treatments received, and how they were handled during model development or evaluation, if relevant | 9-10 |
| *Data preparation* | 7 | D;E | Describe any data pre-processing and quality checking, including whether this was similar across  relevant sociodemographic groups | 9-10 |
| *Outcome* | 8a | D;E | Clearly define the outcome that is being predicted and the time horizon, including how and when assessed, the rationale for choosing this outcome, and whether the method of outcome assessment is  consistent across sociodemographic groups | 9-10 |
|  | 8b | D;E | If outcome assessment requires subjective interpretation, describe the qualifications and demographic characteristics of the outcome assessors | 9-10 |
|  | 8c | D;E | Report any actions to blind assessment of the outcome to be predicted | 10 |
| *Predictors* | 9a | D | Describe the choice of initial predictors (e.g., literature, previous models, all available predictors) and  any pre-selection of predictors before model building | 11 |
|  | 9b | D;E | Clearly define all predictors, including how and when they were measured (and any actions to blind assessment of predictors for the outcome and other predictors) | 11 |
|  | 9c | D;E | If predictor measurement requires subjective interpretation, describe the qualifications and demographic characteristics of the predictor assessors | 11 |
| *Sample size* | 10 | D;E | Explain how the study size was arrived at (separately for development and evaluation), and justify that  the study size was sufficient to answer the research question. Include details of any sample size calculation | 11 |
| *Missing data* | 11 | D;E | Describe how missing data were handled. Provide reasons for omitting any data | 11 |
| *Analytical methods* | 12a | D | Describe how the data were used (e.g., for development and evaluation of model performance) in the analysis, including whether the data were partitioned, considering any sample size requirements | 11-12 |
|  | 12b | D | Depending on the type of model, describe how predictors were handled in the analyses (functional form,  rescaling, transformation, or any standardisation). | 11 |
|  | 12c | D | Specify the type of model, rationale^2^, all model-building steps, including any hyperparameter tuning,  and method for internal validation | 11-12 |
|  | 12d | D;E | Describe if and how any heterogeneity in estimates of model parameter values and model performance was handled and quantified across clusters (e.g., hospitals, countries). See TRIPOD-Cluster for  additional considerations^3^ | NA |
|  | 12e | D;E | Specify all measures and plots used (and their rationale) to evaluate model performance (e.g., discrimination, calibration, clinical utility) and, if relevant, to compare multiple models | 11-12 |
|  | 12f | E | Describe any model updating (e.g., recalibration) arising from the model evaluation, either overall or for particular sociodemographic groups or settings | NA |
|  | 12g | E | For model evaluation, describe how the model predictions were calculated (e.g., formula, code, object, application programming interface) | 11-12 |
| *Class imbalance* | 13 | D;E | If class imbalance methods were used, state why and how this was done, and any subsequent methods to  recalibrate the model or the model predictions | 12 |
| *Fairness* | 14 | D;E | Describe any approaches that were used to address model fairness and their rationale | NA |
| *Model output* | 15 | D | Specify the output of the prediction model (e.g., probabilities, classification). Provide details and  rationale for any classification and how the thresholds were identified | 12 |
| *Training versus*  *evaluation* | 16 | D;E | Identify any differences between the development and evaluation data in healthcare setting, eligibility  criteria, outcome, and predictors | 13,  Table 1 |
| *Ethical approval* | 17 | D;E | Name the institutional research board or ethics committee that approved the study and describe the participant-informed consent or the ethics committee waiver of informed consent | 9 |

| **OPEN SCIENCE** | | | | |
| --- | --- | --- | --- | --- |
| *Funding* | 18a | D;E | Give the source of funding and the role of the funders for the present study | 24 |
| *Conflicts of interest* | 18b | D;E | Declare any conflicts of interest and financial disclosures for all authors | 24 |
| *Protocol* | 18c | D;E | Indicate where the study protocol can be accessed or state that a protocol was not prepared | NA |
| *Registration* | 18d | D;E | Provide registration information for the study, including register name and registration number, or state  that the study was not registered | 9 |
| *Data sharing* | 18e | D;E | Provide details of the availability of the study data | 24 |
| *Code sharing* | 18f | D;E | Provide details of the availability of the analytical code^4^ | 24 |
| **PATIENT & PUBLIC INVOLVEMENT** | | | | |
| *Patient & Public Involvement* | 19 | D;E | Provide details of any patient and public involvement during the design, conduct, reporting, interpretation, or dissemination of the study or state no involvement. | NA |
| **RESULTS** | | | | |
| *Participants* | 20a | D;E | Describe the flow of participants through the study, including the number of participants with and without the outcome and, if applicable, a summary of the follow-up time. A diagram may be helpful. | 13 |
|  | 20b | D;E | Report the characteristics overall and, where applicable, for each data source or setting, including the key dates, key predictors (including demographics), treatments received, sample size, number of outcome events, follow-up time, and amount of missing data. A table may be helpful. Report any  differences across key demographic groups. | 13 |
|  | 20c | E | For model evaluation, show a comparison with the development data of the distribution of important predictors (demographics, predictors, and outcome). | 13,  Suppl Tab. 1 |
| *Model development* | 21 | D;E | Specify the number of participants and outcome events in each analysis (e.g., for model development, hyperparameter tuning, model evaluation) | 13 |
| *Model specification* | 22 | D | Provide details of the full prediction model (e.g., formula, code, object, application programming interface) to allow predictions in new individuals and to enable third-party evaluation and implementation, including any restrictions to access or re-use (e.g., freely available, proprietary)^5^ | 13-16 |
| *Model performance* | 23a | D;E | Report model performance estimates with confidence intervals, including for any key subgroups (e.g., sociodemographic). Consider plots to aid presentation. | 13-16 |
|  | 23b | D;E | If examined, report results of any heterogeneity in model performance across clusters. See TRIPOD  Cluster for additional details^3^. | NA |
| *Model updating* | 24 | E | Report the results from any model updating, including the updated model and subsequent performance | 13-16 |
| **DISCUSSION** | | | | |
| *Interpretation* | 25 | D;E | Give an overall interpretation of the main results, including issues of fairness in the context of the  objectives and previous studies | 17-19 |
| *Limitations* | 26 | D;E | Discuss any limitations of the study (such as a non-representative sample, sample size, overfitting, missing data) and their effects on any biases, statistical uncertainty, and generalizability | 17-19 |
| *Usability of the model in the context of current care* | 27a | D | Describe how poor quality or unavailable input data (e.g., predictor values) should be assessed and handled when implementing the prediction model | 17-19 |
|  | 27b | D | Specify whether users will be required to interact in the handling of the input data or use of the model,  and what level of expertise is required of users | 17-19 |
|  | 27c | D;E | Discuss any next steps for future research, with a specific view to applicability and generalizability of  the model | 19 |

**Legend.** 1. D=items relevant only to the development of a prediction model; E=items relating solely to the evaluation of a prediction model; D;E=items applicable to both the development and evaluation of a prediction model. 2. Separately for all model building approaches. 3. TRIPOD-Cluster is a checklist of reporting recommendations for studies developing or validating models that explicitly account for clustering or explore heterogeneity in model performance (eg, at different hospitals or centres). Debray et al, BMJ 2023; 380: e071018 [DOI: 10.1136/bmj-2022-071018].

From: Collins GS, Moons KGM, Dhiman P, et al. BMJ 2024;385:e078378. doi:10.1136/bmj-2023-078378.

***Supplementary Table 3. Sensitivity analyses***

***A] Train (mean CV)***

|  | ***Accuracy*** | ***AUROC*** | ***PR-AUC*** |
| --- | --- | --- | --- |
| *PICU* |  |  |  |
| *KNN* | *0.75* | *0.81* | *0.79* |
| *MICE* | *0.75* | *0.81* | *0.79* |
| *Inotropes* |  |  |  |
| *KNN* | *0.80* | *0.88* | *0.82* |
| *MICE* | *0.81* | *0.88* | *0.82* |
| *Mechanic ventilation* |  |  |  |
| *KNN* | *0.83* | *0.85* | *0.64* |
| *MICE* | *0.83* | *0.85* | *0.64* |
| *Death* |  |  |  |
| *KNN* | *0.95* | *0.85* | *0.34* |
| *MICE* | *0.94* | *0.83* | *0.35* |

***[B] Test (95%CI)***

|  | ***Accuracy*** | ***AUROC*** | ***PR-AUC*** |
| --- | --- | --- | --- |
| *PICU* |  |  |  |
| *KNN* | *0.78 (0.74-0.83)* | *0.85 (0.82-0.89)* | *0.84 (0.79-0.89)* |
| *MICE* | *0.77 (0.74-0.81)* | *0.85 (0.81-0.88)* | *0.86 (0.82-0.89)* |
| *Inotropes* |  |  |  |
| *KNN* | *0.87 (0.83-0.90)* | *0.93 (0.90-0.95)* | *0.88 (0.83-0.92)* |
| *MICE* | *0.86 (0.82-0.89)* | *0.93 (0.90-0.95)* | *0.88 (0.83-0.93)* |
| *Mechanic ventilation* |  |  |  |
| *KNN* | *0.85 (0.82-0.87)* | *0.85 (0.81-0.90)* | *0.66 (0.59-0.75)* |
| *MICE* | *0.85 (0.82-0.88)* | *0.86 (0.82-0.90)* | *0.68 (0.60-0.76)* |
| *Death* |  |  |  |
| *KNN* | *0.95 (0.94-0.96)* | *0.86 (0.78-0.92)* | *0.34 (0.17-0.53)* |
| *MICE* | *0.95 (0.94-0.96)* | *0.89 (0.81-0.96)* | *0.43 (0.26-0.66)* |

*95% CI were calcualed using 2000 bootstrap replicates. KNN = k-nearest neighbour imputation, MICE = multivariate imputation by chained equations.*

^15^**The REKAMLATINA-3 MIS-C Study Group Investigators** ^ǂ^

Kathia Luciani (Hospital de Especialidades Pediátricas Omar Torrijos Herrera; Ciudad de Panamá, Panamá), Mariana Fabi (Hospital de Niños Sor María Ludovica, Ciudad de La Plata; Buenos Aires, Argentina), Graciela Espada, Marcela Álvarez (Hospital de Niños Ricardo Gutiérrez; Buenos Aires, Argentina), Martha I Álvarez-Olmos (Fundación Cardioinfantil IC; Bogotá, Colombia), Jaime Fernández-Sarmiento (Fundación Cardioinfantil IC/Universidad de la Sabana; Bogotá, Colombia), Paola Pérez-Camacho (Fundación Valle del Lili & Departamento de Pediatría, Facultad de Ciencias de la Salud, Universidad Icesi; Cali, Colombia), Saulo Duarte-Passos (Hospital Universitario de Faculdade de Medicina de Jundiai; Sao Paolo, Brazil), Maria C Cervi (Faculdade de Medicina de Ribeirāo Preto, Universidad de Sāo Paolo; Sao Paolo, Brazil), Edwin M Cantillano (Hospital Regional del Norte, Instituto Hondureño de Seguridad Social; San Pedro de Sula, Honduras), Beatriz A Llamas-Guillén (Hospital del Niño Morelense, Cuernavaca; Morelos, México), Mónica Velásquez-Méndez (Hospital Universitario San Vicente Fundación; Medellín, Colombia), Patricia Saltigeral-Simental (Star Médica Hospital Infantil Privado e Instituto Nacional de Pediatría; Ciudad de México, México), Enrique Chacon-Cruz (Hospital General de Tijuana; Tijuana, México & Think Vaccines LLC; Houston, Texas; USA), Miguel García-Domínguez (Hospital Pediátrico de Sinaloa “Dr. Rigoberto Aguilar Pico; Sinaloa, México), Karla L Borjas Aguilar (Hospital María, Especialidades Pediátricas e Instituto Hondureño de Seguridad Social, Hospital de Especialidades; Tegucigalpa, Honduras), Ana V Villarreal-Treviño (Hospital Regional Materno Infantil de Alta Especialidad, Monterrey; Nuevo León, México), Sandra Beltrán, Andrea Gatica (Hospital Juan Pablo II, Ciudad Guatemala, Guatemala), Fernanda Cofré (Hospital Roberto del Río, Santiago, Chile), Virgen Gómez (Centro Médico Universidad Central del Este (UCE) & Hospital Infantil “Dr. Robert Reid Cabral, Santo Domingo, Dominican Republic), Heloisa HS Marques (Hospital Das Clinicas da Faculdade Medicina da USP, Sao Paolo, Brasil), Nadina E Rubio-Pérez (Hospital Universitario Dr. José Eleuterio González, Universidad Autónoma de Nuevo León, Nuevo León, México), Luis M Garrido-García (Hospital Ángeles de las Lomas, Ciudad de México, México), Luisa B Gámez-González (Hospital Infantil de Especialidades de Chihuahua, Chihuahua, México), Carlos Daza (Hospital Materno Infantil José Domingo De Obaldía, Chiriquí, Panamá), Genara M Santana-Chalas (Hospital Regional Universitario Dr. Arturo Grullón (Santiago, Dominican Republic), Humberto García-Aguilar (Centro Médico Nacional 20 de Noviembre ISSSTE, Ciudad de México, México), Elmer H Zapata-Yarlequé (Clínica San Felipe & Hospital Cayetano de Heredia, Lima, Perú), Lucila Martínez-Medina (Centenario Hospital Miguel Hidalgo, Aguas Calientes, México), Adán Cuatecontzi-Romero (Hospital de la Niñez Oaxaqueña “Dr. Guillermo Zarate Mijangos”, Oaxaca, México), Shirley Cuan (Zona Pediátrica Hospital de Niños & Hospital Herrera Llerandi, Ciudad Guatemala, Guatemala), Alejandro Díaz (Hospital General de Medellín, Medellín, Colombia), Adrián Collia (Sanatorio Mater Dei, Buenos Aires, Argentina), Lorena Franco (Hospital Infantil Municipal de Córdoba, Córdoba, Argentina), Elizabeth Assandri (Hospital Pediátrico Centro Hospitalario Pereira Rossell & Hospital CASMU (Centro de Asistencia del Sindicato Médico del Uruguay, Montevideo, Uruguay), Adriana Díaz-Maldonado (Fundación Hospital Pediátrico La Misericordia (HOMI), Bogotá, Colombia), Elizabeth Castaño, Ximena Norero, Raúl Esquivel, Jacqueline Levy, Katherina Miranda, Scarlett Sinesterra, Manuel Alvarado, Aldo Campos (Hospital del Niño Dr. José Renán Esquivel; Ciudad de Panamá, Panamá), Adriana Yock-Corrales, Alejandra Soriano-Fallas, Kattia Camacho-Badilla, Jéssica Gómez-Vargas (Hospital Nacional de Niños “Dr. Carlos Sáenz Herrera”, Centro de Ciencias Médicas, Caja Costarricense de Seguro Social (CCSS), San José, Costa Rica), Lourdes Dueñas (Hospital Nacional de Niños Benjamín Bloom, San Salvador, El Salvador), Pilar Guarnizo, Manuel Huertas-Quiñones, Diana C Medina-Ramos, Sara I Aguilera-Martínez, Verónica Morales-Burton, (Fundación Cardioinfantil IC, Bogotá, Colombia), Jaime Patiño, Lina M Sandoval-Calle (Fundación Valle del Lili & Universidad Icesi, Cali, Colombia), Rolando Andrés Paternina-de la Ossa (Universidad de Sāo Paolo, Sao Paolo, Brazil), Diana López-Gallegos (Star Médica Hospital Infantil Privado, Ciudad de México, México), Juan Pablo Rojas (Fundación Clínica Infantil Club Noel; Cali, Colombia), Elizabeth Moreno (University of California, San Diego, CA, USA), Mónica Pujadas, Maria C Pirez (Hospital Pediátrico Centro Hospitalario Pereira Rossell, Montevideo, Uruguay), Fernando García-Rodríguez (Hospital Universitario Dr. José Eleuterio González, Universidad Autónoma de Nuevo León, Nuevo León, México), Martha Márquez-Aguirre (Instituto Nacional de Pediatría, Ciudad de México, México), Tirza de León (Hospital Materno Infantil José Domingo De Obaldía, Chiriquí, Panamá), Jesús G Montaño-Durón (Hospital General de Tijuana, Tijuana, México), Manuel Munaico-Abanto (Clínica San Felipe, Lima, Perú), Daniel Jarovsky (Santa Casa de São Paolo School of Medical Sciences, São Paulo, Brasil), Maynor G Bravo-López, Alejandro Ellis (Sanatorio Mater Dei, Buenos Aires, Argentina), Antonio González-Mata (Hospital Pediátrico Universitario “Agustín Zubillaga”, Barquisimeto, Venezuela), Mario Melgar, Antonio Luévanos-Velázquez (Antiguo Hospital Civil de Guadalajara Fray Antonio Alcalde, Guadalajara, Jalisco, México.
